# Deciphering treatment resistance in metastatic colorectal cancer: roles of drug transports, EGFR mutations, and HGF/c-MET signaling

**DOI:** 10.3389/fphar.2023.1340401

**Published:** 2024-01-10

**Authors:** Najah Albadari, Yang Xie, Wei Li

**Affiliations:** College of Pharmacy, University of Tennessee Health Science Center, Memphis, TN, United States

**Keywords:** metastatic colorectal cancer, resistance, anti-EGFR, anti-MET, ABC drug transporters, tyrosine kinase inhibitors

## Abstract

In 2023, colorectal cancer (CRC) is the third most diagnosed malignancy and the third leading cause of cancer death worldwide. At the time of the initial visit, 20% of patients diagnosed with CRC have metastatic CRC (mCRC), and another 25% who present with localized disease will later develop metastases. Despite the improvement in response rates with various modulation strategies such as chemotherapy combined with targeted therapy, radiotherapy, and immunotherapy, the prognosis of mCRC is poor, with a 5-year survival rate of 14%, and the primary reason for treatment failure is believed to be the development of resistance to therapies. Herein, we provide an overview of the main mechanisms of resistance in mCRC and specifically highlight the role of drug transports, EGFR, and HGF/c-MET signaling pathway in mediating mCRC resistance, as well as discuss recent therapeutic approaches to reverse resistance caused by drug transports and resistance to anti-EGFR blockade caused by mutations in EGFR and alteration in HGF/c-MET signaling pathway.

## 1 Introduction

In the United States, colorectal cancer (CRC) is the third most frequently diagnosed cancer and the third most common cause of cancer death in men and women (Siegel et al., 2023). Notably, during the past decades, the incidence of CRC in people younger than 50 years has gradually increased by 1%–2% annually, with a more advanced stage (Siegel et al., 2023). Given that approximately 30% of CRCs are associated with hereditary factors, no single risk factor could account for most cases of CRC (Brenner et al., 2014). The well-established risk factors may include smoking, obesity, high-fat or caloric diet, as well as lack of physical activity (Brenner et al., 2014). Currently, CRC is managed by a multi-model based treatment strategy, including surgery, radiotherapy, traditional chemotherapy, and the recently developed immunotherapy and targeted therapy (Dekker et al., 2019). However, due to the heterogeneity and complexity of CRC itself, there is still an unmet need to achieve a satisfactory overall survival (OS), which is evidenced by the fact that 50% of CRC patients will ultimately develop metastatic disease with a median survival of 2–3 years (Nielsen et al., 2014; Krasteva and Georgieva, 2022). The primary locations where metastasis from CRC to specific sites frequently occurs are lymph nodes, liver, lungs, and peritoneum (Riihimaki et al., 2016).

**FIGURE 1:**
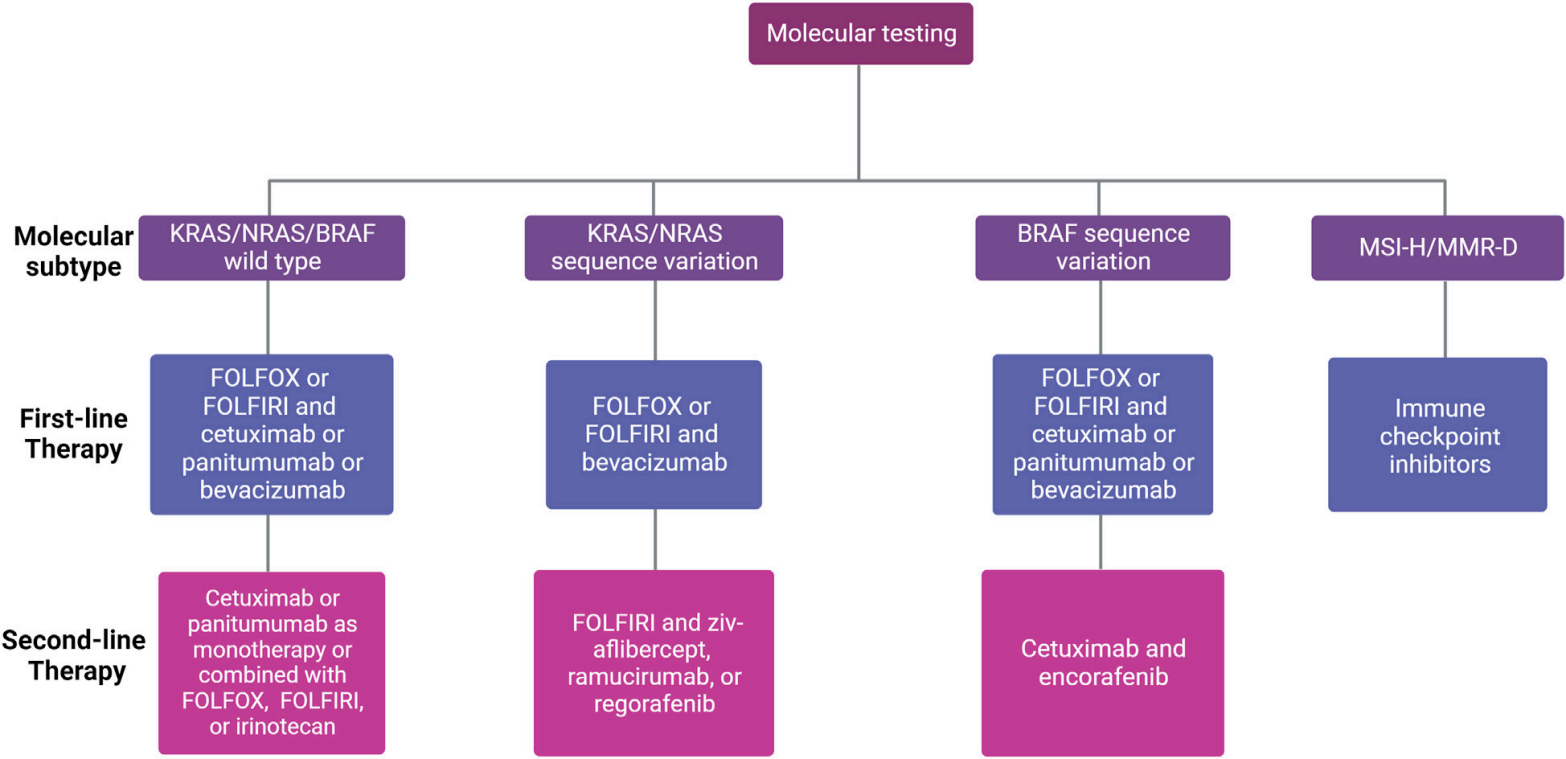
First- and second-line treatment for patients with unresectable mCRC according to molecular subtype. Different factors influence the choice of first-line therapy for mCRC, including existing comorbidities, age of the patient, tumor location (left *versus* right), and molecular profile (RAS/BRAF and microsatellite instability (MSI) status). The choice of second-line therapy depends on the systemic therapies given in the first-line setting.

Currently, recommended treatments for local-regional CRCs include surgical resection, embolization to block the tumor blood supply, and radiotherapy (Young et al., 2014; Seager et al., 2021; Gogoi et al., 2022). However, due to the existence of circulating tumor cells and the related satellite metastasis, adjuvant systemic therapy is of potential importance to improve OS (Basch et al., 2023; Yu et al., 2023). Once metastasis has been identified, the commonly applied adjuvant treatments include chemotherapy, targeted therapy, and immunotherapy (Figure 1). Also, radiation therapy presents a viable treatment option for CRC patients with oligometastasis in the liver and lungs (Tam and Wu, 2019; Mohamad et al., 2022) For chemotherapy, the standard treatment regimen includes fluoropyrimidines (either intravenous fluorouracil (5-FU) or oral capecitabine) in combination with oxaliplatin (OX, DNA alkylating agent) or irinotecan (DNA topoisomerase I (TOP 1) inhibitor) (FOLFOX or CAPOX) or (FOLFIRI or CAPIRI) regimens, and a combination drug in a pill form called TAS-102 or Lonsurf (trifluridine and tipiracil) (Prager et al., 2023). According to the current guidelines, the folic acid derivative, leucovorin together with 5-FU combined with oxaliplatin (FOLFOX) or 5-FU combined with irinotecan (FOLFIRI) are recommended as the first-line therapy for patients with mismatch repair proficient (pMMR), microsatellite stable (MSS) metastatic CRC (mCRC) (Morris et al., 2023). Oxaliplatin-based regimens (FOLFOX or CAPOX) or irinotecan-based regimens (FOLFIRI or CAPIRI) plus the vascular endothelial growth factor (VEGF) inhibitor bevacizumab as an alternative option in first-line treatment for patients with advanced colon cancer (Cervantes et al., 2023). Bevacizumab has been identified to extend the OS of mCRC together with trifluridine-tipiracil treatment (Prager et al., 2023). Other than that, aflibercept, also known as ziv-aflibercept, ramucirumab, and regorafenib were also approved by the FDA as the anti-VEGF antibodies to treat CRC (Grothey et al., 2013; Ohishi et al., 2023). Cetuximab and panitumumab are well-developed monoclonal antibodies (mAbs) targeting epidermal growth factor receptor (EGFR) that have been approved by the FDA, which are used to treat mCRC patients with RAS wild type (WT) (Van Cutsem et al., 2016). Cetuximab, which inhibits EGFR, plus encorafenib, a small molecule BRAF inhibitor, could also improve the progression-free survival (PFS) for CRC patients with a BRAF V600E mutation (Tabernero et al., 2021). Notably, because systemic administration of chemotherapeutic drugs could cause numerous adverse effects, immunotherapy targeting cancer-specific markers is attracting more attention. Thus, due to the expression of the immunosuppressive molecules blocking antitumor immunity, programmed cell death-1 (PD-1) and cytotoxic T lymphocyte-associated protein 4 (CTLA-4), on the surface of activated immune cells, the immune checkpoint inhibitors, anti-PD-1 such as pembrolizumab and nivolumab, and anti-CTLA-4 antibodies ipilimumab were also approved by FDA only for patients with microsatellite instability-high/mismatch repair deficient (MSI-H/MMR-D) mCRC and exhibited multiple efficacies in CRC patients (Myint and Goel, 2017). However, in the setting of MSS or pMMR, which comprises 95% of mCRC (Biller and Schrag, 2021), multiple actively recruiting clinical trials (Table 1) are currently evaluating combining immune checkpoint blockade with other modalities to stimulate an immunogenic response and turn “immune-cold” tumors into “immune-hot” to overcome the resistance of MSS/pMMR mCRC to immunotherapy (Wu et al., 2023). It should be noted that this strategy has not been successful in evoking immunogenic response in any phase 3 clinical trials (Eng et al., 2019; Tabernero et al., 2022). Also, hundreds of clinical trials are currently active or recruiting mCRC patients to test the safety and efficacy of new drugs and new combinations with the ultimate objective of further improving the OS of mCRC patients. Selected trials are summarized in Table 1.

**TABLE 1:** Selected examples of ongoing clinical trials for mCRC.

Title	Combination	Phase	Status	NCT ID
Sotorasib and panitumumab *versus* investigator’s choice for participants with Kirsten Rat Sarcoma (KRAS) p.G12C mutation (CodeBreak300)	Sotorasib and panitumumab *versus* TAS-102 or regorafenib	Phase III	Active, not recruiting	NCT05198934
Phase 3 study of MRTX849 with cetuximab vs. chemotherapy in patients with advanced colorectal cancer with KRAS G12C mutation (KRYSTAL-10)	MRTX849 and cetuximab *versus* mFOLFOX6 or FOLFIRI	Phase III	Recruiting	NCT04793958
A study of tucatinib with trastuzumab and mFOLFOX6 *versus* standard of care treatment in first-line HER2^+^ metastatic colorectal cancer (MOUNTAINEER-03)	Tucatinib, trastuzumab, and mFOLFOX6 *versus* FOLFOX6 given with or without either cetuximab or bevacizumab	Phase III	Recruiting	NCT05253651
Tucatinib combined with trastuzumab and TAS-102 for the treatment of HER2 positive metastatic colorectal cancer in molecularly selected patients, 3T Study	Tucatinib, trastuzumab, and TAS-102	Phase II	Not yet Recruiting	NCT05356897
A study of nivolumab-relatlimab fixed-dose combination *versus* regorafenib or TAS-102 in participants with later-lines of metastatic colorectal cancer (RELATIVITY-123)	Nivolumab and relatlimab fixed-dose combination *versus* regorafenib or TAS-102	Phase III	Recruiting	NCT05328908
Study of XL092 + atezolizumab vs. regorafenib in subjects with metastatic colorectal cancer (STELLAR-303)	XL092 and atezolizumab *versus* regorafenib	Phase III	Recruiting	NCT05425940
Phase II study of pembrolizumab plus capecitabine and bevacizumab in microsatellite stable metastatic colorectal cancer	Pembrolizumab, bevacizumab, and capecitabine	Phase II	Active, not recruiting	NCT03396926
Nivolumab and ipilimumab and radiation therapy in microsatellite stable (MSS) and microsatellite instability (MSI) high colorectal and pancreatic cancer	Nivolumab, ipilimumab, and radiation	Phase II	Recruiting	NCT03104439
Phase 2 study evaluating response and biomarkers in patients with microsatellite stable (MSS) advanced colorectal cancer treated with nivolumab in combination with relatlimab	Nivolumab and relatlimab	Phase II	Recruiting	NCT03642067
Avelumab combined with cetuximab and irinotecan for treatment refractory metastatic colorectal microsatellite stable cancer—A proof of concept, open label non-randomized Phase IIa study. the AVETUXIRI trial	Avelumab, cetuximab, and irinotecan	Phase II	Recruiting	NCT03608046
Phase II trial of Nivolumab and metformin in patients with treatment refractory MSS metastatic colorectal cancer	Nivolumab and metformin	Phase II	Active, not recruiting	NCT03800602
A Study evaluating the safety and efficacy of targeted therapies in subpopulations of patients with metastatic colorectal cancer (INTRINSIC)	• Inavolisib with cetuximab or bevacizumab	Phase I	Recruiting	NCT04929223
	• Atezolizumab, tiragolumab and bevacizumab or atezolizumab and tiragolumab			
	• Atezolizumab and SY-5609			
	• Divarasib and cetuximab			
	• Divarasib, cetuximab and FOLFOX or FOLFIRI			
Testing the combination of two anti-cancer drugs, DS-8201a and AZD6738, for the treatment of patients with advanced solid tumors expressing the HER2 protein or gene, (The DASH Trial)	Trastuzumab deruxtecan (DS-8201a) and with ceralasertib (AZD6738)	Phase I/Ib	Recruiting	NCT04704661
A study of amivantamab monotherapy and in addition to standard-of-care chemotherapy in participants with advanced or metastatic colorectal cancer (OrigAMI-1)	• Amivantamab monotherapy	Phase Ib/II	Recruiting	NCT05379595
	• Amivantamab with mFOLFOX6			
	• Amivantamab with FOLFIRI			
A Phase 1b study of the OxPhos inhibitor ME-344 combined with bevacizumab in previously treated metastatic colorectal cancer	ME-344 and bevacizumab	Phase Ib	Recruiting	NCT05824559
Platform study of JDQ443 in combinations in patients with advanced solid tumors harboring the KRAS G12C mutation	JDQ443 and trametinib or ribociclib or cetuximab	Phase Ib/II	Recruiting	NCT05358249
A safety, tolerability and efficacy study of NC410 plus pembrolizumab in participants with advanced unresectable or metastatic solid tumors	NC410 and pembrolizumab	Phase Ib/II	Recruiting	NCT05572684
First-in-human study of ICT01 in patients with advanced cancer	• ICT01 monotherapy	Phase I/II	Recruiting	NCT04243499
	• ICT01 and pembrolizumab			
COM902 (A TIGIT Inhibitor) in subjects with advanced malignancies	• COM902 monotherapy	Phase I	Recruiting	NCT04354246
	• COM902 and COM701			
	• COM902, COM701, and pembrolizumab			
Phase II, single arm study of neoadjuvant dostarlimab (TSR-042) in stage II and III deficient mismatch repair colon cancers	Dostarlimab	Phase II	Recruiting	NCT05239546
A Phase I/II clinical trial of FOLFOX bevacizumab plus botensilimab and balstilimab (3B-FOLFOX) in patients with MSS metastatic colorectal cancer	FOLFOX, bevacizumab, balstilimab and botensilimab	Phase I/II	Recruiting	NCT05627635
SX-682 and nivolumab for the treatment of RAS-mutated, MSS unresectable or metastatic colorectal cancer (the STOPTRAFFIC-1 Trial)	SX-682 and nivolumab	Phase Ib/II	Recruiting	NCT04599140
A study of fruquintinib in combination with tislelizumab in advanced solid tumors	Fruquintinib and tislelizumab	Phase Ib/II	Recruiting	NCT04577963
Phase I trial of adagrasib (MRTX849) in combination with cetuximab and irinotecan in patients with colorectal cancer	MRTX849, cetuximab, and irinotecan	Phase I	Not yet Recruiting	NCT05722327

As in the cases of conquering many other cancers, treatment resistance, which occurs in 90% of patients with metastatic cancer (Longley and Johnston, 2005), is one of the major problems that oncologists in CRC management confront. Over the past 2 decades, significant progress has been made in comprehending the mechanisms contributing to intrinsic and acquired treatment resistance in mCRC. The main mechanisms of drug resistance in mCRC include disturbance of drug targets, drug metabolism, and drug transportation, and alterations in cell death and carcinogenesis signaling pathways, and tumor immune microenvironment. Numerous thorough and insightful reviews providing a detailed description of these mechanisms have been published, and readers keen on the topic are advised to refer to these excellent reviews (Xie et al., 2020; Wang et al., 2022b; Grassilli and Cerrito, 2022). Furthermore, recently the impact of the angiogenesis fibroblast growth factor receptors (FGFR) signaling pathway has gained attention in CRC (Dienstmann et al., 2014; Luo et al., 2020; Ioffe et al., 2021). Colon cancer cell lines exhibit amplification in FGFR (Mathur et al., 2014; Jiang et al., 2017), and FGFR were found as mediators of colon cancer cell progression (Henriksson et al., 2011). Additionally, the identification of FGFR3 overexpression was found to be related to the unfavorable prognosis of mCRC, implying it is a potential therapeutic target (Fromme and Schildhaus, 2018). Thus, multiple studies are investigating the efficacy of FGFR inhibitors in colorectal cancer, while some have not yet been applied in clinical research (Jiang et al., 2017; Yang et al., 2020a; Yamamoto et al., 2020; Liu et al., 2022).

Given the accumulating literature regarding this field, the present review will briefly pinpoint the aforementioned mechanisms underlying treatment resistance in mCRC. Subsequently, strategies to overcome resistance caused by drug transporters and approaches to reverse anti-EGFR resistance mediated by EGFR mutations in mCRC will be illustrated. Also, we will discuss approaches targeting the c-MET/HGF signaling pathway and their application in mCRC, including preclinical and clinical research, hoping to provide insightful views on fighting against this lethal disease.

## 2 Mechanisms of treatment resistance in mCRC

### 2.1 Disturbance of drug target/metabolism

5-FU needs to be transported into the cells and perform its function by diffusion or highly binding with nucleobase carrier (Griffith and Jarvis, 1996). It is metabolized through anabolic and catabolic pathways (Sethy and Kundu, 2021). In the anabolic route, with the involvement of thymidine phosphorylase (TP), uridine phosphorylase (UP), uridine kinase (UK), orotate phosphribosyl transferase (OPRT) and phosphoribosyl pyrophosphate (PRPP), 5-FU is converted to active metabolites, such as fluorouridine triphosphate (FUTP), fluorouridine diphosphate (FUDP), FUDP-sugars, fluorodeoxyuridine monophosphate (FdUMP) (Longley et al., 2003; Peters, 2020). Then, these fluoronucleotide metabolites exert their function by inhibiting the fundamental target enzyme thymidylate synthase (TS) activity and by RNA and DNA misincorporation, thereby inducing DNA/RNA impairment and cell death (Longley et al., 2003; Sethy and Kundu, 2021). In the catabolic route, 5-FU is converted into dihydro fluorouracil (DHFU) by dihydropyrimidine dehydrogenase (DPD) (mostly in the liver), forming α-fluoro-β- alanine (FBAL) and α-fluoro-β-ureido propionic acid (FUPA) which are excreted through kidneys (Longley et al., 2003). Interruption of 5-FU conversions mentioned above could affect its activation and lead to therapy resistance (Peters, 2020). Among the metabolic enzymes that could potentially affect the activation of 5-FU, the key factors related to 5-FU resistance are TS, DPD, and TP (Sethy and Kundu, 2021). *In vitro*, TS mRNA level was increased in 5-FU resistant CRC cells. Elevated TS expression was reported to be associated with poor OS rate in patients with mCRC (Kamoshida et al., 2004). In addition, acquired 5-FU resistance is identified to be correlated with TS gene amplification and mutations. Therefore, it indicates that once enhanced TS amplification is detected in the tumors, 5-FU may not be an ideal treatment option (Wang et al., 2004; Zhang et al., 2008). Moreover, elevated DPD expression is also considered a major cause of 5-FU resistance (Kornmann et al., 2003; Soong et al., 2008). DPD inhibition, on the other hand, has been shown to re-sensitize CRC to 5-FU both *in vitro* and *in vivo*, indicating that interference of DPD could be an approach to overcome 5-FU resistance (Zhang et al., 2020). TP catalyzes the conversion of 5-FU to its active form, 5-fluoro-2′-deoxyuridine. It has been reported that elevated TP expression is a promising phenotype for 5-FU response and is correlated with prolonged PFS in CRC patients (Lindskog et al., 2014). Furthermore, low activity of UK, UP, uridine 5’-monophosphate synthase (UMPS), and uridine monophosphate kinase (UMPK) as well as OPRT have also been related to 5-FU resistance (Fujii et al., 2003; Humeniuk et al., 2009; Griffith et al., 2013; Peters, 2020). Conversely, high UP and OPRT activity have been validated to contribute to 5-FU sensitivity (Houghton and Houghton, 1983; Schwartz et al., 1985).

Irinotecan inhibits DNA TOP 1, resulting in misalignment and therefore inhibiting DNA replication and transcription (Cuya et al., 2017; Kciuk et al., 2020). Irinotecan can be activated by carboxylesterase 1/2 (CES1/2) and catalyzed to a more active metabolite SN-38, both of which could form an inhibitory complex with TOP1 and DNA (Takano and Sugiyama, 2017). Meanwhile, cytochrome P450 isoforms 3A4 and 3A5 (CYP3A4/5) catalyze the degradation of irinotecan. It has been found that an increase in CYP3A4/5 activity or a decrease in CES1/2 activity is correlated with irinotecan resistance (Trumpi et al., 2015). Uridine diphosphate glucuronosyltransferase 1As (UGT1As) can glucuronidate SN-38 and form an inactive SN-38 glucuronide. Therefore, the genetic polymorphisms of UGT1A have also been related to irinotecan resistance (Takano and Sugiyama, 2017; de Man et al., 2018).

Oxaliplatin is a platinum DNA alkylating reagent widely used in CRC treatment. It has been shown that oxaliplatin could present a cross-resistance with cisplatin and carboplatin (Peters, 2020). Thio-containing molecules, such as glutathione (GSH), have been found to form conjugates with platinum compounds, thereby blocking their activity (Hamaguchi et al., 1993). On the other hand, decreased GSH level was found to enhance oxaliplatin sensitivity (Mohn et al., 2010).

### 2.2 Cell death signaling pathways

Apoptosis is a programmed cell death process. It is a homeostatic mechanism to maintain the cell population. B-cell lymphoma 2 (Bcl-2), a well-established apoptosis marker, has been shown to promote CRC progression and treatment resistance (Ramesh and Medema, 2020). The myeloid cell leukemia 1 (Mcl-1), a pro-survival member of the Bcl-2 protein family, has been reported to confer chemoresistance by translocating to the nucleus (Fu et al., 2022). Inhibitor of apoptosis proteins (IAPs) are also critical for CRC treatment resistance. On the one hand, cellular IAP1 and IAP2 could contribute to the tumor necrosis factor-alpha (TNF-α) induced nuclear factor kappa-B (NF-κB) activation and therefore promote cell proliferation (McCann et al., 2021). On the other hand, an X-linked inhibitor of apoptosis protein (XIAP) could inhibit apoptosis by suppressing caspase3/7 and caspase-9 (Lippa et al., 2007; McCann et al., 2021). Additionally, chemotherapies induce apoptosis by upregulating tumor suppression genes such as p53 (TP53). However, up to 60% of CRC patients harbor the mutated p53, which has been reported to be associated with poor prognosis and chemotherapy resistance (Iacopetta, 2003; Boyer et al., 2004; Robles et al., 2016). On the contrary, CRCs with WT p53 were found to be more sensitive to oxaliplatin and irinotecan treatment (Manic et al., 2003; Weekes et al., 2010). Furthermore, it was reported that tumor cells with mutant p53 were resistant to the cytotoxic effect induced by CD8^+^ T cells (Michel et al., 2021). As a matter of fact, since p53 status was related to reduced immune cell infiltration and programmed death-ligand 1 (PD-L1) expression, p53 mutation has been recognized as a negative indicator for treatment response (Li et al., 2020a).

Autophagy is a catabolic process in which cellular organelles are degraded while the constituent metabolites are recycled to promote cell survival (Mizushima and Komatsu, 2011). Therefore, it is believed that anti-cancer treatment-induced stress could induce autophagy, which in turn contributes to treatment resistance (Smith and Macleod, 2019). In CRC, autophagy markers Beclin-1 and Ras-related in brain 7 (Rab-7) have been correlated with treatment resistance and poor survival (Koustas et al., 2019). Interleukin-6 (IL-6) in the tumor microenvironment (TME) has also been identified to trigger autophagy via the Janus kinase 2/Beclin-1 (JAK2/BECN1) pathway. Specifically, BECN1 Y333 phosphorylation could be adopted as a marker for CRC prognosis and chemotherapy resistance (Hu et al., 2021). Additionally, pharmacological and genetic inhibition of autophagy by inhibiting the vacuolar protein sorting 34 kinase has been demonstrated to induce natural killer (NK) cells and T cells infiltration within the CRC TME and therefore improve survival in a mouse model (Noman et al., 2020). Ferroptosis is a unique pattern of cell death hallmarked by iron-dependent phospholipid peroxidation (Jiang et al., 2021). It has been indicated that Lipocalin 2, an iron homeostasis-regulating protein, is overexpressed in CRC and related to 5-FU treatment resistance (Chaudhary et al., 2021). Nuclear factor erythroid 2-related factor 2 (Nrf2) is a key regulator that plays pivotal roles in lipid peroxidation (Dong et al., 2020). It has been indicated that Nrf2 regulated heme oxygenase-1 (HO-1) activation could promote 5-FU treatment resistance in CRC (Kang et al., 2014).

### 2.3 Tumor microenvironment

TME is composed of various infiltrated tumor-associated cells and the cytokines secreted by those cells. In CRC, TME is shaped specifically for tumor progression and treatment resistance (Bruijns et al., 2014; Chen et al., 2021). As one of the critical components of TME, cancer-associated fibroblasts (CAF) have been identified to promote CRC resistance and relapse in multiple ways. Proteomic analysis has revealed that CAF-derived C-C motif chemokine ligand 2 (CCL-2) induced the upregulation of FGFR4 expression in CRC cells. Elevated FGFR4 accumulation activates the β-catenin pathway, further contributing to the 5-FU and oxaliplatin resistance (Liu et al., 2013). Meanwhile, CAF activity is also regulated by proinflammatory factors such as interleukin-1β (IL-1β) and transforming growth factor β (TGFβ). It has been indicated that IL-1β and TGFβ could recruit CAF to the TME (Guillén Díaz-Maroto et al., 2019), which subsequently induces the carcinogenesis pathways within CRC cells, including JAK/STAT, PI3K/Akt, and activates hedgehog transcription factor GLI2, leading to cancer stem cells (CSCs) proliferation and intrinsic chemotherapy resistance (Tang et al., 2018; Guillén Díaz-Maroto et al., 2019; Linares et al., 2023). In addition, CAF could secrete a variety of tumor-nourishing cytokines. CAF-activated interleukin 6/8 (IL-6/8)/JAK pathway has been related to the stabilization of bromodomain and extra-terminal protein family member 4 (BRD4), resulting in the resistance to bromodomain and extra-terminal motif (BET) inhibitors (Wang et al., 2021b). CAF-derived interleukin 17A (IL-17A) was reported to promote CRC cancer-initiating cells renewal and tumor growth (Lotti et al., 2013). Meanwhile, CAF-secreted exosomes have been identified to convey micro-RNAs (miRNA) that could contribute to tumor metastasis and chemotherapy resistance (Hu et al., 2019; Zhang et al., 2021a).

Another subset of tumor infiltration cells within the CRC TME is the myeloid-derived suppressor cells (MDSCs). MDSCs are mainly composed of immature granulocytic cells and monocytic cells (Wang et al., 2020). It has been observed that MDSC could inhibit T-cell function by secreting arginase I, which is essential for T-cell proliferation. In addition, MDSC also impairs the function of NK cells by producing nitric oxide (Rodriguez et al., 2004; Toor et al., 2016; Stiff et al., 2018). Therefore, MDSCs contribute to the immunotherapy resistance in CRCs. It has been demonstrated that CRCs bearing KRAS G12D mutation repressed the expression of interferon regulatory factor 2 (IRF2), which in turn induced the expression of CXC motif chemokine ligand 3 (CXCL3). CXCL3 binds with the CXC chemokine receptor type 2 (CXCR2) receptors on MDSCs, promoting their migration toward the TME. This mechanism is highly correlated with the resistance to anti-PD1 therapy (Liao et al., 2019). By targeting a receptor of TNF-related apoptosis-inducing ligand (TRAIL) that was highly expressed on MDSCs, a significantly induced CD8^+^ T-cell infiltration within the TME was observed, simultaneously with the depletion of MDSCs (Tang et al., 2022).

Tumor-associated macrophages (TAM) are also actively involved in the TME regulation. MDSCs together with proinflammatory cytokines could polarize TAM from the anti-tumor M1 into the pro-tumor M2 subtype (Sun et al., 2023). In CRCs, mutant KRAS positively correlated with the infiltration of TAM. Such gain-of-function mutation of KRAS in CRC cells reprogrammed TAM by secreting tumor-derived colony-stimulating factor 2 and lactate to promote tumor progression as well as develop resistance to EGFR inhibitors such as cetuximab (Liu et al., 2021). In addition, TAM-derived ornithine decarboxylase has been identified to be related to 5-FU resistance (Zhang et al., 2016).

### 2.4 Epigenetic mechanisms

#### 2.4.1 DNA methylation

DNA methylation is an epigenetic change that induces the covalent transfer of methyl groups to DNA and thus affects the modulation of the transcription, suppressing expression (Romero-Garcia et al., 2020). As one of the crucial epigenetic phenomena, it occurs in the cytosine of CpG dinucleotide island, driving the progression of CRC and chemoresistance (Patnaik and Anupriya, 2019). DNA methylation has been proven to confer resistance to multiple chemotherapy treatments such as 5-FU, irinotecan, and oxaliplatin. CpG-methylation was found to contribute to the downregulation of miR34a, which results in the upregulation of colony-stimulating factor 1 receptor (CSF1R) expression and resistance to 5-FU in CRC (Shi et al., 2020). Additionally, researchers applied the reduced representation bisulfite sequencing (RRBS) in irinotecan and oxaliplatin-resistant cell lines and found DNA methylation contributed to the aberrant regulation of Alu elements, which was important for chemoresistance (Lin et al., 2015). Furthermore, MDSCs were reported to be regulated by DNA methylation through an IRF8-independent mechanism, resulting in the accumulation of MDSCs and the progression of CRC (Smith et al., 2020).

#### 2.4.2 Non-coding RNAs

Non-coding RNAs (ncRNAs) could transcript without protein-coding potential. miRNA and long noncoding RNAs (lncRNAs) are known RNAs that participate in CRC resistance. For instance, in the cetuximab-resistant CRC cells, approximately 280 ncRNA transcripts were found to be downregulated (Jing et al., 2019).

miRNAs were found to regulate the drug resistance by affecting the apoptosis. For instance, overexpression of miR-21 in CRC was found to display effects on decreasing 5-FU-induced G2/M cell cycle arrest as well as apoptosis, which correlates with 5-FU resistance (Valeri et al., 2010). MiR-23a is related to the microsatellite instability (MSI) of CRC. The ectopic expression of miR-23a was elevated in MSI CRC cells or tissues compared to the MSS CRC cells. Ectopic expression of miR-23a increases the survival of CRC cells but reduces the viability and promotes cell apoptosis when miR-23a was downregulated upon being treated with 5-FU (Li et al., 2015). MiR-10b was proven to inhibit the pro-apoptotic BIM, a Bcl-2 family member, and results in the chemoresistance of 5-FU in CRC cells (Nishida et al., 2012). MiR-520g was shown to confer resistance of 5-FU and oxaliplatin-induced apoptosis both *in vitro* and *in vivo*. Furthermore, it was found to mediate the resistance by downregulating the p21 expression (Zhang et al., 2015). Furthermore, miR-100 and miR-125b were overexpressed in cetuximab-resistant CRC cells and repressed Wnt/β-catenin negative regulators, leading to the upregulation of Wnt signaling pathway and CRC treatment resistance (Lu et al., 2017). Meanwhile, the repression of miRNA expression also promotes chemoresistance. MiR-181a, miR-135a, and miR-302c were found to be repressed mediated by DNA methylation and promote the microsatellite unstable CRC development as well as 5-FU resistance (Shi et al., 2018).

LncRNAs are noncoding RNAs longer than 200 nucleotides. It has been demonstrated that lncRNAs were participated in each stage of gene expression, such as transcription, posttranscription, RNA editing, and cell cycle regulation (Li et al., 2014). LncRNAs have also been reported to regulate CRC resistance by affecting several key mechanisms such as ABC transporters and multiple signaling pathways. For instance, Histone H3 methylation and deacetylation were found to promote the elevation of colorectal cancer-associated lncRNA (CCAL), followed by the activation of Wnt/β-catenin signaling pathway and ultimately the increase of ABCB1 expression (Ma et al., 2016). LncRNA CACS15 was discovered to be upregulated in oxaliplatin resistance of CRC cells by regulating the miR-145/ABCC1 axis (Gao et al., 2019). LncRNA miR100HG was observed to be overexpressed in acquired cetuximab resistance in CRC. Researchers found miR100HG conferred cetuximab resistance by regulating Wnt signaling (Lu et al., 2017). Furthermore, lncRNA nuclear-enriched abundant transcript 1 (NEAT1) was discovered to activate Wnt signaling by interacting with DEAD-Box Helicase 5 (DDX5) protein and contribute to CRC progression (Hirukawa et al., 2019). Lnc-RP11-536 K7.3 were found to promote proliferation and oxaliplatin resistance in CRC via SRY-Box Transcription factor 2 (SOX2)/Ubiquitin Specific Peptidase 7(USP7)/HIF-1α signaling axis (Li et al., 2021). lncRNA ELFN1 antisense RNA 1 (ELFN1-AS1) has been identified to mediate the downregulation of Meis Homeobox 1 (MEIS1) gene via the Enhancer of Zeste 2 Polycomb Repressive Complex 2 (EZH2)/DNA (cytosine-5)-methyltransferase 3A (DNMT3a) axis and promote the oxaliplatin resistance in CRC (Li et al., 2022). In addition, research has shown that CCALs could promote oxaliplatin resistance by transferring from CAFs to the cancer cells by exosomes secretion, which ultimately suppresses the CRC cell apoptosis and induces CRC resistance (Deng et al., 2020). Additionally, CAFs were found to promote CRC resistance by transferring lncRNA H19 which activated the β-catenin pathway by competing with the miR-141 that inhibited the stemness of CRC cells (Ren et al., 2018).

### 2.5 Alterations of carcinogenesis signaling pathways

#### 2.5.1 EGFR families

**FIGURE 2:**
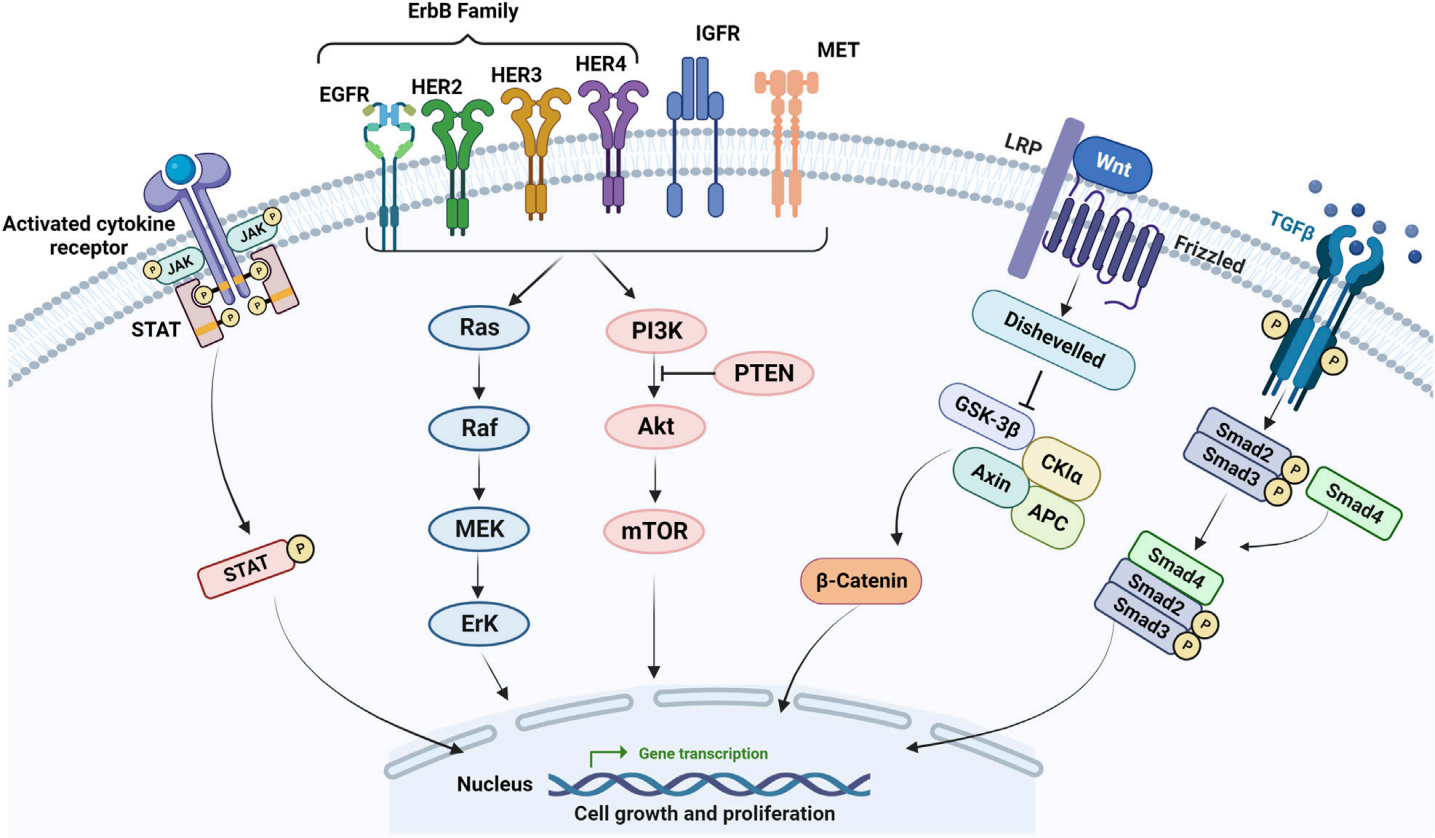
Main signaling pathways involved in mCRC resistance. Activating mutations in the EGFR and its downstream effectors such as RAS, BRAF, and PIK3CA, the loss of PTEN, activation of RAS/MAPK, PIK3/Akt, Wnt/β-catenin, JAK/STAT, TGF-β/SMAD pathways as well as IGF-1 molecular pathway correlated with the progression of CRC and resistance to chemotherapy and anti-EGFR in mCRC.

EGFR is a transmembrane glycoprotein and belongs to the ErbB family, which includes EGFR (ErbB-1), HER2 (ErbB-2), HER3 (ErbB-3), and HER4 (ErbB4) (Ohishi et al., 2023). Among these receptors, EGFR is the most investigated and is involved in multiple cellular pathways such as PI3K/Akt/PTEN/mTOR and RAS/RAF/MEK/ERK (Figure 2) (Liu et al., 2018). Overactivation of EGFR pathways has been observed in multiple malignant tumors including CRC. Therefore, therapies targeting EGFR and its downstream targets have been adopted for CRC treatment (Cully et al., 2006; Meriggi et al., 2009).

RAS/RAF/MEK/ERK is a canonical pathway that transmits the extracellular signals to the intracellular targets. The extracellular-single-regulated kinases (ERK) is the most important subfamily of mitogen-activated protein kinases (MAPK) for cell proliferation (Fang and Richardson, 2005). By regulating cell growth, survival, and differentiation, ERK/MAPK was considered crucially involved in regulating cell metabolism (Degirmenci et al., 2020). Notably, 40% of the CRC cases harbor KRAS mutation (Yu et al., 2022). 10%–15% of CRC has been identified with BRAF mutation (Corcoran et al., 2012). Specifically, KRAS and BRAF mutations were so prevalent in mCRC that it is attributed to the resistance of anti-EGFR agents such as cetuximab and panitumumab (Li et al., 2020b). On the other hand, because RAS activates the downstream RAF/MEK/ERK cascade, the blockage of MEK has been previously considered as an alternative strategy to overcome anti-EGFR resistance (Santarpia et al., 2012; Yu et al., 2022). However, the observed MEK inhibitors (MEKi) outcomes were not as promising as expected. It could be related to the reactivation of receptor tyrosine kinases (RTKs) following the MEK inhibition, which is regulated by growth factor receptor-bound protein 7-polo-like kinase-1 (GRB7-PLK1) and ultimately leads to the resistance to MEK inhibitors (Yu et al., 2022). In addition, the MEK/ERK pathway also contributes to the expansion of PD-L1-promoted colorectal CSCs. PD-L1 is well-known for assisting the escape of tumor cells from immune surveillance. By interacting with high mobility group AT-hook 1 (HMGA1), PD-L1 activation could induce MEK/ERK and PI3K/Akt pathways to facilitate CSC expansion (Wei et al., 2019).

PI3K/Akt/mTOR is another crucial pathway downstream of EGFR and is closely related to CRC progression and treatment resistance. PI3K is a heterodimeric molecule that is activated by EGFR signaling. Akt is a serine/threonine protein kinase (Ser/Thr kinase) situated downstream of PI3K and mediates the effects of PI3K on tumor progression. mTOR is the mammalian target of rapamycin, which is downstream of Akt and promotes protein translation, growth, and angiogenesis (Johnson et al., 2010). PI3K/Akt activation has been reported in 60%–70% of CRC patients (Malinowsky et al., 2014). Mutation of the PIK3CA gene and the loss of PTEN (the negative regulator of PI3K/Akt pathway) have been identified as the potential causes for the resistance to anti-EGFR agents in CRC (Chalhoub and Baker, 2009; Sanaei et al., 2022). Other than that, PI3K/Akt signaling also conferred cetuximab resistance by mediating the loss of mismatch repair gene mutL homolog 1 (MLH1) (Han et al., 2020). In addition, activation of PI3K/Akt signaling was found to mediate the 5-FU resistance by the upregulation of HIF-1α (Dong et al., 2022).

Members of the human epidermal growth factor receptor (HER) are related to cancer cell differentiation, proliferation as well as migration (Paul and Hristova, 2019; Ye et al., 2022). HER2 (ERBB2) amplification and mutation are frequently correlated with EGFR-targeted therapy resistance (Suwaidan et al., 2022). Amplification of HER2 has been found in around 3% of patients with mCRC and 5% of patients with KRAS and NRAS WT tumors (Strickler et al., 2022). Researchers also revealed that mCRC patients with elevated HER2 expression demonstrated poor responses to anti-EGFR therapy such as cetuximab (Bertotti et al., 2011), which is related to lung metastases and poor OS (Sartore-Bianchi et al., 2019). HER3 (ERBB3) is also expressed aberrantly in CRC (Yonesaka, 2021). It was proved that HER3 could bind to the neuregulin family which consists of EGF-like family ligands and induce HER3 activation. Furthermore, HER3 has six docking sites for the p85 subunit of PI3K, which facilitate the binding of HER3 and PI3K, followed by the activation of the PI3K/Akt pathway (Kawakami and Yonesaka, 2016). In cetuximab resistant mCRC samples, higher HER3 expression was associated with poor OS. Thus, HER3 has been considered as a predictive marker to evaluate cetuximab treatment response (Cushman et al., 2015). HER4 (ERBB4) was found to bind with HER3 ligands and EGFR ligands and contribute to CRC progression (Ye et al., 2022). HER4 expression was upregulated in aggressive CRC cell lines. It enhances CRC growth via Ras and WNT signaling (Williams et al., 2015). Furthermore, HER4 expression has been associated with worse outcomes such as lymph node metastasis through epithelial–mesenchymal transition (EMT) (Jia et al., 2020).

#### 2.5.2 Wnt/β-catenin

The Wnt/β-catenin pathway is correlated with cell survival, proliferation, differentiation as well as migration (Neiheisel et al., 2022). After binding with its receptors LRP5/6, Wnt activates the cytoplasmic protein Disheveled (DVL) and suppresses the complex of glycogen synthase kinase 3β (GSK3β)/CK1α/AXIN/adenomatous polyposis (APC). Thereby, it inhibits the degradation of β-catenin. The stabilized β-catenin could subsequently translocate to the nucleus and bind with T-cell factor/lymphoid enhancer factor (TCF/LEF) transcription factors and induce the following gene transcription, which is vital for carcinogenesis (Zhang and Wang, 2020). The dysregulation of this pathway has been identified to promote CSCs renewal, cell proliferation, and differentiation, and is closely related to CRC progression and treatment resistance (Yamamoto et al., 2022; Zhao et al., 2022). Wnt/β-catenin induced CRC treatment resistance has been attributed to mainly three aspects: highly metastatic CSCs, regulation of non-coding RNA, and TME regulation (Zhao et al., 2022). For instance, doublecortin-like kinase 1 (DCLK1), a cancer stem cell marker could confer the 5-FU resistance in CRC via the aberrant activation of Wnt/β-catenin signaling (Wang et al., 2022a). Cetuximab resistance was found to be related to the upregulation of miR-199b-3p via the activation of the Wnt/β-catenin pathway (Han et al., 2022). CAF was reported to induce the increase of miR-92a-3p in CRC cells, followed by the activation of the Wnt/β-catenin pathway, enhanced cell stemness, EMT, CRC metastasis, and 5-FU resistance (Hu et al., 2019). Besides, 5-FU resistance in CRC was also associated with the increased HIF-1α expression via the aberrant activation of Wnt/β-catenin signaling (Dong et al., 2022).

#### 2.5.3 TGF-β

TGF-β actively regulates tumor migration, invasion, EMT, angiogenesis, and therapy resistance (Maslankova et al., 2022). In the TGF-β/SMAD pathway, the binding of TGF-β with its ligand TβII could recruit and phosphorylate TβRI and mediate the following activation of SMAD2/3. SMAD4 could bind to the activated SMAD2/3 and ultimately regulate gene transcription. The non-SMAD signaling of TGF-β pathway is also vital in regulating tumorigenesis, mediating with the involvement of pathways such as PI3K/Akt/mTOR as well as RAS/RAF/MEK/ERK (Zhang et al., 2021b). In the progression of CRC, TGF-β switches its role from tumor suppressor to tumor promoter (Chiavarina et al., 2021). It has been reported that TGF-induced EMT could increase the stemness and PD-L1 expression on cancer cells, contributing to 5-FU resistance in CRC (Soundararajan et al., 2019; Jiang and Zhan, 2020). TGF-β could also be activated by the mediator protein complex subunit 12 (MED12), which may upregulate the mesenchymal marker expression and induce chemotherapy resistance in CRC (Brunen et al., 2013). TGF-β has also been shown to cooperate with micro-RNA and mediate OX resistance in CRC. By cooperating with MiR-34a, TGF-β has been reported to inhibit macro-autophagy and induce oxaliplatin resistance (Sun et al., 2017a).

#### 2.5.4 JAK/STAT

JAK/STAT signaling pathway is involved in the control of stem cell maintenance and inflammatory responses (Thomas et al., 2015) and is crucial for cell migration, growth as well as differentiation (Wang et al., 2021a). Upon activation, JAKs become docking sites for STATs, thereby assisting the translocation of STATs from the cytoplasm to the nucleus to initiate transcription (Slattery et al., 2013). Activation of the JAK/STAT pathway was correlated with the progression of CRC. STATs could upregulate the expression of apoptosis inhibitors (BCL-xa Mcl-1), cell cycle regulators (cyclins D1/D2, c-Myc), and inducers of angiogenesis (VEGF) (Buettner et al., 2002). For instance, aberrant activation of JAK/STAT could downregulate cytoplasmic polyadenylation element binding proteins (CPEBs) 3 and induce CRC cell proliferation, migration, and invasion (Fang et al., 2020). Researchers found that JAK2/STAT3 signaling was activated in radioresistant CRC tissues and induced radioresistance by elevating cyclin D2 (CCDN2) expression, which resulted in the persistent growth of CSCs, intact cell cycle and proliferation as well as low levels of DNA damage accumulation (Park et al., 2019). In addition, STAT3 expression was reported to be positively related to the resistance of 5-FU-based chemoradiotherapy (Spitzner et al., 2014). Besides, protein tyrosine phosphatase receptor type D (PTPRD), a phosphatase in JAK/STAT signaling, was closely related to Bevacizumab resistance due to its deleterious mutations (Hsu et al., 2018).

#### 2.5.5 IGFs

The insulin-like growth factors (IGFs) are mitogens that regulate key physiological processes such as cell proliferation, differentiation, and apoptosis mediated by the interaction of IGF-1 receptor (IGF-1R) (Yu and Rohan, 2000). IGF-1R has been validated as the upstream of EGFR and is involved in multiple pathways including Ras/Raf/MAP kinase and PI3K/Akt (Hu et al., 2008; Scartozzi et al., 2010). Increasing evidence shows that the IGF-1 molecular pathway also promotes tumor metastasis, treatment resistance, and poor prognosis in malignant tumors including CRC (Yu and Rohan, 2000; Wu et al., 2002; Sun et al., 2017b). For instance, a clinical study showed CRC patients with KRAS WT and positive IGF-1 have worse responses than negative IGF-1 patients to cetuximab and irinotecan treatments (Scartozzi et al., 2010). Researchers also discovered that IGF-1/IGF-1R polymorphisms could be the prognostic/predictive markers for the efficacy of cetuximab in mCRC patients with WT KRAS (Winder et al., 2010). IGF-binding protein-3 (IGFBP3) was found to be a modulator of IGF-signaling and able to inhibit tumor cell invasion. Additional evidence showed that matrix metallopeptidase (MMP7) in CRC cells mediates the IGFBP3 degradation and results in the IGF-dependent CRC progression (Gallego et al., 2009; Massoner et al., 2009).

## 3 Drug transporters

Drug transporters play dynamic roles in regulating the absorption, distribution, metabolism, and excretion of drugs. Besides, they are also actively involved in the multidrug resistance (MDR) when they are activated to pump drugs out of the cells (Wang et al., 2022b). In general, there are two major superfamily membrane transporters that contain more than 400 types of drug transporters: the adenosine triphosphate (ATP)-binding cassette (ABC) transporters and solute carrier (SLC) transporters (Giacomini et al., 2010). The ABC transporter superfamily includes 48 membrane proteins that could be divided into 7 sub-families, from ABC-A to ABC-G (Moore et al., 2023). The MDR in CRC is mainly attributed to the overexpression of ABC transporters, resulting in decreased intracellular drug concentration and diminished therapeutic efficacy (Ceballos et al., 2019). P-glycoprotein (P-gp), encoded by the MDR1/ABCB1 gene, is a phosphoglycoprotein. Overexpression of the P-gp protein was extensively found in CRC cell lines and specimens that were resistant to 5-FU, doxorubicin, and oxaliplatin (Zhou et al., 2017; Du et al., 2018; Wang et al., 2022b). In addition, P-gp has also been reported to pump irinotecan and metabolites out of the cells (Trumpi et al., 2015). High expression of P-gp has been detected at the time of CRC diagnosis, correlating with inherent resistance in several colon cancer cell lines to anti-cancer drugs (Fojo et al., 1987; Spoelstra et al., 1991; Meschini et al., 2000). ABC sub-family A transporters, a group of well-known cholesterol transporters, are also related to CRC resistance. ABC-A transporters have been identified to regulate EMT and induce chemoresistance in CRC (Alketbi et al., 2023). It has been reported that increased ABCA2, ABCA1, and ABCA5 expressions were found in chemo-resistance CRC cell lines. When inhibiting the activity of ABCA5, it may re-sensitize the chemo-resistant CRC cells to 5-FU and oxaliplatin treatment (Ravindranathan et al., 2019). Multidrug resistance proteins (MRPs) belong to the C family of ABC transporters (Sodani et al., 2012). Overexpression of MRP2 has been identified in platinum-resistant cancer cell lines (Taniguchi et al., 1996). MRP8/ABCC11 and ABCC5 have been reported to confer 5-FU resistance by transporting the active monophosphorylated metabolites in CRC (Pratt et al., 2005). In addition, FOXM1, as the key transcription factor, was reported to bind to the promoter of ABCC10 and induce 5-FU resistance in CRC cells. FOXM1 and/or ABCC10 inhibition could sensitize resistant CRC cells to 5-FU (Xie et al., 2017). Furthermore, FOXM1 overexpression has been correlated with EMT induction and P-gp overexpression (Yang et al., 2020b).

### 3.1 Strategies to overcome resistance mediated by drug transporters in mCRC

Due to the crucial role played by ABC transporters in cancer chemoresistance, over the years, extensive efforts have been made to develop therapeutic approaches to block or modulate their activity to increase the concentration of anti-cancer drugs within the cells and re-establish the drug sensitivity of resistant cancer cells (Kathawala et al., 2015). In this context, different approaches to counteract the activity of ABC transporters in tumor cells have been reported in the literature which include inhibition of the efflux function of the transporters, downregulation of the expression of the transporters by arresting the transcription factors regulating their expression, or blockade of the transporter-induced signaling pathways (Kathawala et al., 2015). Numerous small molecules ABC transporters inhibitors or modulators directed mainly against P-gp have been developed. However, none of these inhibitors have been approved in clinical trials mainly because they exhibit either high toxicities and low specificity or poor therapeutic efficacy (Ozols et al., 1987; Ferry et al., 1996; Baer et al., 2002; Rubin et al., 2002; Seiden et al., 2002; Friedenberg et al., 2006; Gandhi et al., 2007; Kelly et al., 2011). These include first-generation MDR modulators (such as verapamil, cyclosporine A, and quinine), second-generation MDR modulators (such as valspodar and biricodar), and third-generation MDR modulators (such as elacridar, laniquidar, zosuquidar, and tariquidar). Researchers also exploited natural products to develop the fourth generation of ABC transporter inhibitors. These inhibitors show promise for use with chemotherapeutic agents, serving as effective chemosensitizers specifically targeting cancer cells exhibiting MDR (Karthikeyan and Hoti, 2015; Martins-Gomes and Silva, 2023). For instance, the natural compound S-Adenosylmethionine (AdoMet) was reported to reverse the 5-FU-induced upregulation of P-gp in CRC cell lines, HCT 116 p53^+/+^ and LoVo (Mosca et al., 2021). Furthermore, the antitumor efficacy of oxaliplatin was enhanced by ursolic acid, a pentacyclic triterpenic acid found in various natural plants. This enhancement was achieved by downregulating the mRNA and protein levels of P-gp and ABCG2, demonstrated in both *in vitro* and *in vivo* models of CRC using HCT8 and SW480 cells (Zhang et al., 2018). The doxorubicin resistance in P-gp-overexpressing human colon carcinoma cells, SW620 Ad300, was also reversed by phenylpropanoid piperazines derived from a fungal isolate from the gastrointestinal tract of the Australian marine-derived Mugil mullet (Mohamed et al., 2020). Also, the polyhydroxy-flavonoid quercetin (Que) has been shown to significantly improve doxorubicin toxicity in SW620/Ad300 cells via inhibiting the expression of the glutamine transporter solute sarrier family 1, member 5 (SLC1A5) which in turn significantly downregulated the ATP-driven efflux activity of P-gp, leading to the intracellular accumulation of doxorubicin (Zhou et al., 2020).

**FIGURE 3:**
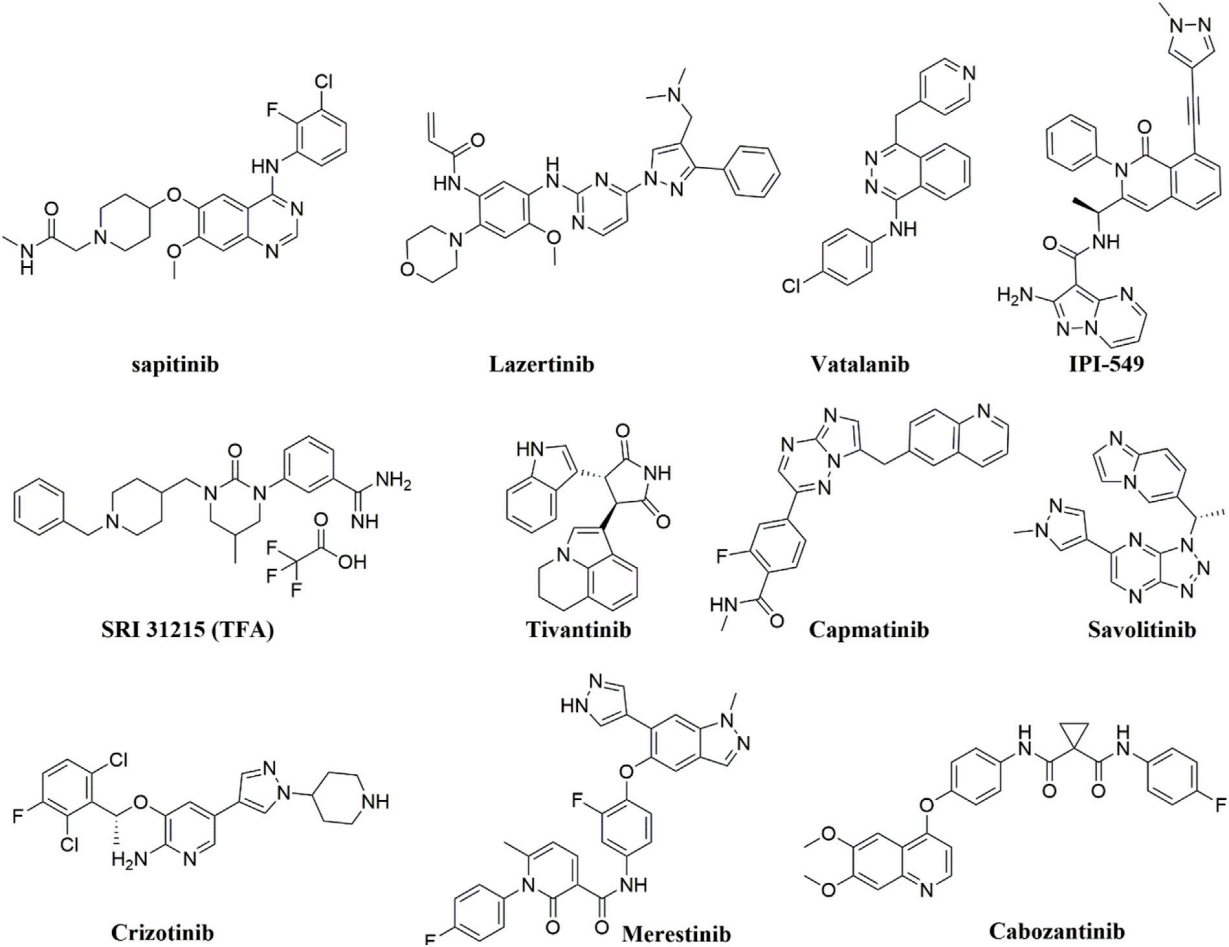
The chemical structures of compounds reviewed in here.

Tyrosine kinase inhibitors (TKIs) have been reported to act as inhibitors of ABC transporters and chemosensitizers in MDR that enhance the therapeutic efficacy of standard chemotherapeutic agents by combination therapy (Wu and Fu, 2018). The structures of compounds reviewed in this review are shown in Figure 3. For instance, it has been reported that imatinib, nilotinib, and ponatinib reversed the P-gp-, ABCG2-, and ABCC10-mediated MDR by inhibiting their efflux activity (Kathawala et al., 2015; Wu and Fu, 2018). Lapatinib was also reported to significantly reduce the MDR mediated by the overexpression of P-gp, ABCC1, ABCG2, and ABCC10 by inhibiting their transport function (Dai et al., 2008; Kuang et al., 2010; Ma et al., 2014) whereas afatinib reversed the chemoresistance mediated by ABCG2 but not P-gp or ABCC1 by suppressing the expression and the function of ABCG2 *in vitro* and *in vivo* (Wang et al., 2014). Regorafenib was also reported to reverse the P-gp- (Wang et al., 2017b) and ABCG2-mediated MDR (Zhang et al., 2019) *in vitro* in CRC cell line SW620/Ad300 (overexpressing P-gp) and S1-M1-80 (overexpressing ABCG2), respectively. Regorafenib did not affect the expression level and cellular localization of P-gp and ABCG2 but attenuated the efflux activity of the transporters and interfered with their ATPase activity (Wang et al., 2017b; Zhang et al., 2019). *In vivo*, regorafenib combined with either paclitaxel or topotecan showed an additive antitumor effect on the growth of the parental colorectal SW620 tumors and S1 tumors, respectively. However, in the SW620/Ad300 (P-gp-overexpressing) and S1-M1-80 (ABCG2-overexpressing) colon cancer xenograft models, the combination of regorafenib and paclitaxel or the combination of regorafenib and topotecan resulted in a synergistic antitumor effect which demonstrated that regorafenib overcame the resistance of paclitaxel to P-gp-overexpressing SW620/Ad300 tumors and the resistance of topotecan to ABCG2-overexpressing S1-M1-80 tumors in mice (Wang et al., 2017b; Zhang et al., 2019).

Gao et al. reported that **sapitinib**, a non-selective inhibitor of EGFR that inhibits both HER2 and HER3 (Barlaam et al., 2013) could reverse MDR to the P-gp substrates, paclitaxel and doxorubicin. This resulted in increased efficacy in P-gp-overexpressing doxorubicin-selected SW620/Ad300 cells and HEK293 cells transfected with the P-gp gene *in vitro* (Gao et al., 2020). In the SW620/Ad300 cells, the addition of 1 µM of sapitinib resulted in a 13- and 100-fold increase in the potency of paclitaxel and doxorubicin, respectively, similar to the inhibitor of P-gp, verapamil. Furthermore, when 5 µM of sapitinib was used, it boosted the potency of paclitaxel by even more (162-fold) compared to the non-resistant parental cell line, SW620. Also, the incubation of HEK293/ABCB1 cells with 1 and 5 µM of sapitinib significantly increased the cytotoxic efficacy of paclitaxel by 14- and 25-fold, respectively, compared to the parental cells HEK293/pcDNA3.1. Sapitinib reversed P-gp-mediated resistance to paclitaxel and doxorubicin by inhibiting the function but not the expression or subcellular localization of the P-gp protein. *In vitro*, sapitinib increased the intracellular accumulation of [^3^H]-paclitaxel in SW620/Ad300 cells compared to SW620/Ad300 cells incubated with the vehicle and to the parental SW620 cell, which do not overexpress the P-gp transporter. Furthermore, sapitinib stimulated the ATPase activity of the P-gp transporter in a concentration-dependent manner (Gao et al., 2020).

Fan et al. reported that **Lazertinib**, the recently approved EGFR TKI, specifically potentiated the efficacy of the MDR transporters substrates, mitoxantrone and topotecan in the mitoxantrone-resistant ABCG2-overexpressing colon carcinoma cell line S1-M1-80 but not in the parental sensitive colon carcinoma S1 (Fan et al., 2022). Lazertinib blocked the drug efflux function of ABCG2 and significantly increased the intracellular accumulation of doxorubicin, a known substrate of ABCG2 in the resistant S1-M1-80 cells in a dose-dependent manner. In contrast, the accumulation of doxorubicin in the parental S1 cells was not affected by lazertinib. Furthermore, the ATPase activity of ABCG2 and P-gp was stimulated by lazertinib; however, their mRNA expression or protein expression levels were not affected by lazertinib treatment in the MDR cells nor their plasma membrane localization in cancer cells. Fan et al. also demonstrated that the MDR reversal effect of lazertinib was unrelated to the inhibition of the EGFR signaling pathway as the expressions of total or phosphorylated Akt and ERK1/2 were not significantly changed in the MDR S1-MI-80 cells (Fan et al., 2022).

Another VEGF TKI, **vatalanib** that failed the Phase III clinical trial in combination with standard FOLFOX chemotherapy in the first-line treatment of patients with mCRC (NCT00056459) (Hecht et al., 2011), was found to inhibit the efflux function of P-gp and ABCG2 in the P-gp and ABCG2-overexpressing colon cancer cells HCT116 and without altering the ABCG2/P-gp mRNA or protein expression levels or the phosphorylation of Akt and ERK1/2. Also, vatalanib sensitized the colon cancer cell lines to SN-38, a substrate of P-gp and ABCG2, under hypoxic growth conditions (To et al., 2015). Noteworthy is that several TKIs were also shown to behave as substrates of ABC transporters at low concentrations and to modulate the expression of some ABC transporters directly or indirectly (Beretta et al., 2017). For instance, continuous exposure (up to 100 days) of the human colon adenocarcinoma cells Caco2 with imatinib (10 μM) was reported to cause an upregulation of the expression of ABCG2 (maximal ∼17-fold) and P-gp (maximal ∼5-fold) and ∼50% reduction in the intracellular accumulation of imatinib (Burger et al., 2005). The fact that TKIs can function either as inhibitors or substrates for ABC transporters necessitates thorough research into TKI chemosensitizing effects. This research could offer a compelling basis for effectively combining TKIs with standard therapy to combat chemoresistance in CRC.

The phosphoinositide 3-kinase gamma isoform (PI3Kγ) inhibitor, **IPI-549** (eganelisib) could also reverse the P-gp-mediated MDR in SW620/Ad300 cells and sensitize SW620/Ad300 cells to P-gp substrates, doxorubicin, vincristine, paclitaxel, and colchicine (De Vera et al., 2019). *In vitro*, IPI-549 increased the intracellular concentration of [^3^H]-paclitaxel by inhibiting the efflux of paclitaxel out of SW620/Ad300 cells and stimulating P-gp-mediated ATPase activity. Exposing SW620/Ad300 cells to 10 μM IPI-549 for more than 72 h did not cause a significant change in the expression of P-gp, and the subcellular localization of P-gp on the cellular membrane remained unchanged (De Vera et al., 2019). *In vivo*, IPI549 improved the anti-tumor efficacy of paclitaxel in tumor xenograft model of P-gp-overexpressing SW620/Ad300 colon cancer with a ratio of growth inhibition for tumor volume (IRV) = 74% and for tumor weight (IRW) = 72%, compared to 42% IRV and 37% IRW for the paclitaxel alone treatment (De Vera et al., 2019). IPI-549 is now under investigation in clinical trials in triple-negative breast cancer (TNBC), renal cell cancer (RCC) as well as head and neck squamous cell carcinoma.

## 4 Alterations in EGFR

Low expression of EGFR or its key low-affinity ligands, amphiregulin (AREG) and epiregulin (EREG) causes loss of target for anti-EGFR therapy in CRC (Jacobs et al., 2009; Seligmann et al., 2016). Several clinical studies have found that low expression levels of AREG and EREG are related to the low response rate to cetuximab in KRAS-WT CRC patients, but not in CRC patients with KRAS-mutation (Khambata-Ford et al., 2007; Jacobs et al., 2009). Furthermore, mutations in the extracellular domain (ECD) of the EGFR, such as S492R, G465E, R451C, and K467T, may be found in those receiving cetuximab and was responsible for their cetuximab-resistance (Montagut et al., 2012; Arena et al., 2015; Bertotti et al., 2015; Morelli et al., 2015). On the other hand, cells overexpressing EGFR S464L, G465R, and I491M mutants are resistant to cetuximab and panitumumab (Arena et al., 2015). Also, Liao demonstrated that increased methylation at R198 and R200 of the ECD of EGFR by protein arginine methyltransferase 1 (PRMT1) sustained signaling activation in the presence of cetuximab and conferred resistance to cetuximab treatment in both mouse orthotopic CRC xenograft model and CRC patients (Liao et al., 2015).

### 4.1 Strategies to overcome resistance caused by mutations in EGFR in mCRC

A second-generation FDA-approved human EGFR mAb, **necitumumab** has been shown to bind to EGFR that harbors all reported cetuximab- and panitumumab-resistant mutations except for those at position G441 (Bagchi et al., 2018). Necitumumab showed clinical efficacy in a phase II study (NCT00835185) in combination with FOLFOX as a first-line treatment for patients with treatment-naïve, locally advanced, or mCRC with an objective response rate (ORR) of 63.6% and a complete response (CR) observed in 4 out of 44 patients enrolled in the study (Elez et al., 2016). The clinical outcome of first-line necitumumab and FOLFOX regimen was better in CRC patients whose tumors were KRAS-WT compared with those whose tumors harbored KRAS exon 2 mutations (Elez et al., 2016). However, necitumumab is still to be evaluated clinically in CRC tumors with mutated EGFR.

Oligoclonal combination inhibitors that can bind to the mutated ECD of EGFR are new approaches to targeting EGFR that are expected to overcome resistance caused by EGFR ECD mutations. For instance, **MM-151** is an oligoclonal combination of three fully human mAbs (P1X, P2X, and P3X) targeted against distinct, nonoverlapping epitopes on EGFR that overcomes signal amplification driven by both low- and high-affinity EGFR ligands (Kearns et al., 2015). In preclinical studies, MM-151 has been shown to outperform currently approved and investigational mAbs, displaying superior EGFR signal amplification inhibition, enhanced downregulation of the EGFR, and engagement of innate immune responses (Kearns et al., 2015). Napolitano et al. demonstrated that MM-151 has a significantly better antitumor activity as compared to cetuximab in an *in vivo* study in three RAS WT human colon cancer xenografts, SW48, LIM1215, and CACO2 (Napolitano et al., 2017b). Most importantly, the superior activity of MM-151 has also been demonstrated in cetuximab-refractory CRC models with five partial responses (PR) and two stable diseases (SD) in seven mice bearing SW48 or LIM1215 xenografts, respectively, and PR in all seven mice bearing CACO2 xenografts (Napolitano et al., 2017b). Furthermore, MM-151 showed efficacy against CRC tumors derived from patients who develop secondary resistance to EGFR blockade by cetuximab or panitumumab due to mutations in the ECD of EGFR (Arena et al., 2016). Arena et al. also showed that MM-151 is active against all known EGFR ECD mutants and is comparable in efficacy to panitumumab against cetuximab-resistant variants that retain sensitivity to panitumumab, particularly EGFR K467T and S492R (Arena et al., 2016). Moreover, Arena et al. showed that MM-151 decreased and stabilized EGFR ECD mutations in circulating cell-free tumor DNA (ctDNA) of mCRC patients who developed EGFR ECD mutations as a result of treatment with cetuximab or panitumumab (Arena et al., 2016). Preliminary results of the Phase I clinical trial (NCT01520389) of MM-151 alone and in combination with irinotecan in patients with solid tumors including patients with refractory mCRC, suggest an acceptable tolerability profile and provide evidence of objective clinical activity of MM151 (Lieu et al., 2015).

**Sym004** is another mixture of two chimeric human–mouse mAbs (futuximab and modotuximab), recognizing nonoverlapping epitopes on EGFR ECD III (Koefoed et al., 2011). Sym004 is superior to cetuximab and panitumumab in both *in vitro* and *in vivo* preclinical studies in tumors showing EGFR ligand–dependent growth (Pedersen et al., 2010; Napolitano et al., 2017a). Sym004 also demonstrated efficacy in cetuximab-resistant CRC xenograft models harboring mutations in EGFR, S492R EGFR-mutant DCR7, and G465R EGFR-mutant LIM1215 (Sanchez-Martin et al., 2016). Furthermore, Napolitano et al. reported that Sym004 could induce a significant reduction in c-MET, Akt, and MAPK phosphorylation in cetuximab-resistant CRC cancer cells (GEO-CR and SW48-CR) even in the presence of the high-affinity ligand TGFα (Napolitano et al., 2017a). Sym004 has shown clinically meaningful rates of PR (13%) and minor tumor regression (44%) of target lesions in the Phase I trial (NCT01117428) in patients with KRAS WT mCRC and acquired resistance to anti-EGFR inhibitors (Dienstmann et al., 2015). However, although Sym004 monotherapy has decreased EGFR ECD in ctDNA of patients enrolled in Phase II of the study (NCT02083653), it did not improve OS or PFS in an unselected population of patients with mCRC and acquired anti-EGFR resistance (Montagut et al., 2018). It is worth noting that the study identified a ctDNA molecularly defined subgroup of patients (RAS/BRAF/EGFR ECD-mutation negative) for whom prior anti-EGFR therapy had failed and Sym004 had improved OS by 5.5 months (Montagut et al., 2018).

## 5 Alterations in HGF/c-MET signaling pathway

The Hepatocyte growth factor/scatter factor (HGF/SF) and its receptor mesenchymal-epithelial transition factor (c-MET) are also critical in CRC progression, metastasis as well as treatment resistance (Yao et al., 2019). Upon the activation by HGF, the MET receptor stimulates the following signaling cascades such as ERK1/2/MAPK, PI3K/Akt, mTOR, STAT, NF-κB, and subsequently attributes to stem cell survival, proliferation, and organogenesis (Boccaccio and Comoglio, 2006; Comoglio et al., 2008; Della Corte et al., 2014; Joosten et al., 2020). MET was discovered to be overexpressed in primary CRC, found in about 1.7% of all CRCs (Raghav et al., 2016), indicating it could be an important prognostic marker for early-stage invasion and regional disease metastasis (Takeuchi et al., 2003). Functionally, MET has been shown to cross react with EGFR and possibly substitute their activity (Karamouzis et al., 2009). Joosten et al. demonstrated that HGF/MET signaling can fully substitute EGFR signals to maintain and expand normal and neoplastic mouse and human CRC stem cells. Furthermore, they observed that the growth inhibition caused by EGFR inhibition can be fully bypassed by HGF stimulation (Joosten et al., 2017; Joosten et al., 2019). Thus, MET is more frequently related to the resistance of EGFR inhibition; up to 22.6% of CRCs that are refractory to anti-EGFR therapy harbor *MET* amplification (Raghav et al., 2016). For instance, a previous study has proved that the MET amplification could induce cetuximab or panitumumab resistance in metastasis CRC patients without KRAS mutations (Bardelli et al., 2013). TGF-α, as the EGFR ligand, was found to be overexpressed through the EGFR-MET interaction and ultimately contributed to cetuximab resistance in colorectal cancer cells (Troiani et al., 2013). Moreover, CD44, a MET co-receptor and a prime transcriptional target of Wnt signaling and highly expressed by intestinal stem cells and adenoma, could be overexpressed upon the activation of the WNT pathway, which mediated the overexpression of MET. The activation of HGF/MET/CD44 signaling has been reported to promote metastasis CRC resistance to EGFR inhibitors (Joosten et al., 2020). Additionally, upregulated HGF was found to induce cetuximab resistance in CRC cells via the binding with SRY-Box Transcription Factor 8 (SOX8), indicating the crucial role of SOX8/HGF/MET in the CRC therapy resistance (Piao et al., 2022). Preclinical and clinical findings suggested that EGFR/MET activation through HGF-induced cetuximab resistance (Liska et al., 2011; Takahashi et al., 2014; Yonesaka et al., 2015). Recently, Kim et al. reported that PFS and OS were significantly shorter in mCRC patients receiving cetuximab + FOLFIRI who had high baseline plasma HGF levels vs. patients with low baseline HGF group (Kim et al., 2022).

### 5.1 Strategies to target the HGF/c-MET pathway in mCRC

Promising progress has been made to overcome resistance to anti-EGFR therapy via targeting the c-MET/HGF pathway using mAbs against the c-MET receptor and its only ligand HGF as well as selective small molecule c-MET inhibitors and multitargeted small-molecule TKIs. Hu et al. reported that the anti-HGF mAb **ficlatuzumab** significantly inhibited the c-MET activation and rescued the capacity of cetuximab to inhibit the phosphorylation of EGFR in cetuximab-resistant CRC cell lines LM1215, SW48, and DiFi (Hu et al., 2016). **Rilotumumab**, a fully human anti-HGF mAb that neutralizes HGF-dependent c-MET signaling, has been investigated in phase Ib/II trial (NCT00788957) in combination with panitumumab in previously treated patients with KRAS-WT mCRC. The addition of rilotumumab to panitumumab has increased the ORR by 10 percentage points compared with panitumumab alone (31% vs. 21%), however; the improvement in ORR did not translate into significant PFS and OS benefits (Van Cutsem et al., 2014). Furthermore, negative study data have been published for **onartuzumab,** which is a humanized, monovalent anti-c-MET antibody that impedes HGF-MET binding and subsequent activation by HGF and does not affect the intrinsic kinase activity of c-MET (Martens et al., 2006; Merchant et al., 2013). In a Phase II (NCT01418222) study in chemonaïve mCRC patients (Bendell et al., 2013), the addition of onartuzumab to the VEGF inhibitor bevacizumab plus mFOLFOX-6 did not significantly improve efficacy outcomes even in MET-positive populations (Bendell et al., 2017).

Anti-MET mAbs have also been used for drug conjugation, resulting in anti-MET antibody-drug conjugates (ADCs) that are independent of MET gene copy number, instead relying on c-MET target expression for activity. For instance, telisotuzumab vedotin (ABBV-399) and TR1801-ADC are two anti-MET ADCs that have been preclinically validated in *in vitro* and *in vivo* models for CRC. **Telisotuzumab vedotin** composed of the c-MET mAb, ABT-700 conjugated to monomethyl auristatin E (MMAE), a tubulin polymerization inhibitor via a cleavable valine–citrulline linker (Wang et al., 2017a). *In vivo*, telisotuzumab vedotin demonstrated synergistic antitumor potency in combination with FOLFIRI in SW-48 xenograft tumors in mice (Wang et al., 2017a). **TR1801-ADC** was constructed by site-specific conjugation of the DNA-crosslinking agent, pyrrolobenzodiazepine (PBD) tesirine (SG3249) to engineered cysteines on the constant region of the heavy chain of the humanized anti-MET mAb hD12 (Gymnopoulos et al., 2020). In PDX models of CRCs with moderate to high c-MET expression, a single dose of 1 mg/kg of TR1801-ADC resulted in complete tumor regression lasting for a period of 4 weeks, with no indications of tumor recurrence (Gymnopoulos et al., 2020).

**SRI 31215** is a small molecule inhibitor of the trypsin-like serine proteases, matriptase, hepsin, and HGF activator (HGFA), the principal proteases required for HGF activation (Owusu et al., 2016; Venukadasula et al., 2016). SRI 31215 inhibited the activation of the tumor-associated fibroblast, pro-HGF, the inactive precursor of HGF in colon cancer cells, blocked the crosstalk between DU145 colon cancer cells and tumor-associated fibroblasts, prevented fibroblast-dependent EMT and migration of cancer cells, and overcame fibroblast-induced resistance to cetuximab and gefitinib in HGF-producing RKO colon cancer cells (Owusu et al., 2016).

On the other hand, **tivantinib** is a non-ATP competitive that was initially described as a selective small molecule c-MET receptor TKI that can disrupt both ligand-dependent c-MET activity and constitutively active c-MET and inhibit the downstream c-MET effectors (Munshi et al., 2010). However, several studies revealed that independent of c-MET signaling, tivantinib also disrupted microtubule dynamics, induced G2/M arrest and apoptosis, overcame ABC transporter–mediated multidrug-resistant, and acted as a microtubule polymerization inhibitor by binding to tubulin via the colchicine-binding site (Basilico et al., 2013; Aoyama et al., 2014; Calles et al., 2015). A single oral dose of 200 mg/kg of tivantinib inhibited the phosphorylation of constitutive c-MET and the growth of c-MET-dependent human colon HT29 xenograft tumors (Munshi et al., 2010). In Phase I/II combination study with irinotecan and cetuximab in selected patients with KRAS WT locally advanced or mCRC (NCT01075048), the addition of tivantinib did not improve PFS, the primary endpoint (Eng et al., 2016). Nevertheless, in the same study, tivantinib in combination with cetuximab and irinotecan significantly improved ORR (54% vs. 30%), PFS (7.9 vs. 5.8 months), and OS (22.3 vs. 17.6 months) compared with placebo in the subgroup of patients whose tumor was MET-high (Eng et al., 2016). Also, among patients with PTEN-low tumors, the addition of tivantinib to cetuximab and irinotecan significantly improved ORR (58% vs. 18%), PFS (11.1 vs. 5.3 months) and OS (25.1 vs. 8.3 months) compared with placebo (Eng et al., 2016). The results seen in patients with MET-high tumors align with findings from Phase II and III clinical trials in patients with advanced hepatocellular carcinoma (HCC) and non-squamous non-small cell lung cancer (NSCLC), respectively, that showed significant PFS and OS benefits associated with tivantinib in patients with MET-high tumors (Santoro et al., 2013; Scagliotti et al., 2015). Combining tivantinib and cetuximab in patients with EGFR-resistant MET-high KRAS WT mCRC in Phase II trial (NCT01892527) suggested efficacy with 10% of patients achieving objective response, including 3 confirmed PR and 1 confirmed CR (Rimassa et al., 2019).

**Capmatinib** is an ATP-competitive and highly selective small molecule c-MET receptor TKI that demonstrated inhibition of MET activation in cancer cells whose growth is driven by MET amplification, MET overexpression, MET exon 14 skipping mutations, and HGF-mediated activation (Baltschukat et al., 2019). *In vitro*, capmatinib sensitized CRC cells to cetuximab in the presence of HGF by abrogating the effect of HGF on EGFR and MET downstream signaling pathways (Kim et al., 2022). In the Phase Ib dose escalation study (NCT02205398), 6 out of 13 c-MET positive mCRC patients whose tumors have become resistant to anti-EGFR treatment treated with capmatinib plus cetuximab achieved stable disease (disease control rate (DCR) of 46.2%) (Delord et al., 2020). However, the study was temporarily halted before initiating the dose expansion part, due to difficulties in identifying patients who met the eligibility criteria.

**Savolitinib** is a selective and potent c-MET inhibitor that showed synergistic tumor inhibition in c-MET amplified CRC PDX preclinical models in combination with the anti-VEGF inhibitor apatinib (Chen et al., 2018). Recently, Gu and others reported that savolitinib significantly decreased the viability and suppressed primary and secondary sphere formation by ABHD5-knockdown CRC cells, HCT116 (Gu et al., 2021). *In vivo,* savolitinib significantly inhibited tumor growth of subcutaneous ABHD5-knockdown HCT116 xenograft models (Gu et al., 2021). The intracellular lipolytic factor, a/b-hydrolase domain-containing 5 (ABHD5), has been identified as a novel tumor suppressor in CRC, and its loss significantly promotes CRC tumorigenesis and metastasis (Ou et al., 2014). Gu et al. showed that loss of ABHD5 promotes YAP-induced c-MET overexpression which in turn increases and sustains the stemness of CRC cells and promotes the development and progression of CRCs (Gu et al., 2021). c-MET inhibition using savolitinib significantly suppressed sphere formation by the patient-derived CRC cells with low ABHD5 and high MET expression, indicating that inhibiting c-MET is an effective strategy for eradicating the CRC stem cell compartment, characterized as low ABHD5 and high c-MET expression (Gu et al., 2021). Also, savolitinib significantly suppressed the HGF-stimulated expansion of human CRC stem cells into organoids (Joosten et al., 2019). In a first-in-human Phase I study (NCT01773018) in patients with locally advanced or metastatic solid tumors, savolitinib showed signs of clinical efficacy in CRC tumor type; one MET-amplified CRC patient achieved SD as a best response (Gan et al., 2019). A phase II study (NCT03592641) evaluated the efficacy of savolitinib in heavily treated patients with RAS WT and MET-amplified mCRC, however; the study was terminated due to inadequate accrual rate with only five patients were eligible for enrollment.

Bardelli and others showed that the multi-kinase inhibitor, **crizotinib** could overcome primary and secondary resistance to anti-EGFR caused by MET amplification in patient-derived CRC xenografts (Bardelli et al., 2013). Also, crizotinib was found to sensitize cetuximab-resistant KRAS mutant CRC cell lines LoVo, HCT116, and DLD1 to radiation therapy (Cuneo et al., 2019). Cuneo et al. showed that pretreatment of cetuximab-resistant KRAS mutant CRC cell lines with crizotinib blocked radiation-induced c-MET phosphorylation, attenuated radiation-induced Akt activation, increased radiation-induced DNA damage, and prolonged DNA double-strand break repair (Cuneo et al., 2019). Interestingly, by seeding a human CRC cell line (HCA-7) in a three-dimensional (3D) culture system in type-I collagen as single cell suspension, Li et al. generated *de novo* and acquired cetuximab resistant CRC cell lines, SC and CC-CR, respectively, that are WT for KRAS, BRAF, PIK3CA, and EGFR, and not amplified MET, but with increased MET/RON tyrosine phosphorylation (Li et al., 2017; Graves-Deal et al., 2019). Crizotinib can overcome cetuximab resistance in these cells, and the combination of crizotinib and cetuximab significantly inhibited the 3D colony growth of SC and CC-CR and the SC subcutaneous xenograft growth and decreased phosphorylation of MET tyrosine, ERK1/2, and Akt (Li et al., 2017; Graves-Deal et al., 2019). Furthermore, both SC and CC-CR are also resistant to panitumumab and MM-151; however, crizotinib can restore panitumumab and MM-151 sensitivity in SC and CC-CR (Graves-Deal et al., 2019). Furthermore, Lev and others reported that crizotinib synergizes with the DNA crosslinker, mitomycin C *in vitro* against CRC cells HT-29, HCT-116, SW-480, and DLD-1 and *in vivo* in HT-29 xenograft model, indicating a promising combination approach for the treatment of advanced CRC (Lev et al., 2017). Moreover, crizotinib can synergistically increase the MEK-inhibitor AZD6244-induced apoptosis and growth inhibition *in vitro* and *in vivo* in KRAS-mutated CRC cells (Van Schaeybroeck et al., 2014). Schaeybroeck et al. demonstrated that MEK inhibitors block the activity of the ERK-dependent metalloprotease ADAM17 (Van Schaeybroeck et al., 2011), which subsequently enhances c-MET-JAK1/2-STAT3 signaling that is essential for the survival of KRAS-mutated CRC but not WT KRAS (Van Schaeybroeck et al., 2014). Thus, targeting both MEK (binimetinib) and c-MET (crizotinib) was evaluated in CRC patients with RAS mutant or RAS WT and aberrant c-MET in a completed Phase I clinical trial (NCT02510001), however; final results from this trial are awaited.

**Merestinib**, another MET kinase inhibitor that can also inhibit other receptor tyrosine kinases has just finished its Phase Ia/b study (Saleh et al., 2020) in combination with ramucirumab in patients with mCRC previously treated with oxaliplatin and/or irinotecan. The combination of merestinib plus ramucirumab was tolerable and the overall disease control rate was 52% with approximately 50% of patients achieving SD. The median PFS was 3.3 months, and the median OS was 8.9 months (Saleh et al., 2020).

**Cabozantinib** is a promiscuous inhibitor of MET tyrosine kinase that has undergone extensive testing in CRC. Cabozantinib also targets receptor tyrosine kinases central to cancer cell growth and tumor angiogenesis, including RET, AXL, TIE2, and VEGFR2 (Song et al., 2015). Similar to crizotinib, cabozantinib, as reported by Deal and others and measured by 3D colony growth and activation state of MET, RON, ERK1/2 can overcome *de novo* and acquired resistance to cetuximab in SC and CC-CR cells mentioned earlier (Graves-Deal et al., 2019). Also, cabozantinib showed significantly greater tumor growth inhibition, significantly reduced tumor vascularity, and glucose uptake, and significantly increased autophagy in CRC PDX mouse models when compared to regorafenib (Scott et al., 2018). Although both cabozantinib and regorafenib have antiangiogenic properties via VEGFR2 and TIE2 inhibition and share similar kinase target profiles, regorafenib does not inhibit MET, which could explain the observed differences in treatment effects of cabozantinib and regorafenib in CRC cells. The efficacy and safety of cabozantinib have been tested in a Phase II study (NCT03542877) in heavily pretreated patients with refractory mCRC. Among the 40 patients evaluable for response, 18 patients met the 12-week PFS primary endpoint, with one patient achieving a PR, and 27 patients achieving SD as the best response (Scott et al., 2022). The ORR and the DCR were 2.5% and 70%, respectively and the median PFS and the median OS were 3.0 and 8.3 months, respectively. Patients with RAS WT mCRC experienced improved PFS and OS compared to patients with RAS mutant mCRC; PFS and OS for RAS WT patients were 4.9 and 10.4 months, respectively, and PFS and OS for RAS mutant mutant patients were 2.7 and 7.0 months, respectively (Scott et al., 2022). Also, a Phase Ib/II clinical trial (NCT02008383) evaluated cabozantinib with or without panitumumab in chemo-refractory, KRAS WT mCRC patients who had received prior treatment with fluoropyrimidine, oxaliplatin, irinotecan, and bevacizumab. Strickler et al. reported that 4 of 25 patients treated with cabozantinib plus panitumumab had PRs, including one patient with EGFR-resistant disease, and the median PFS observed in the EGFR refractory population and median OS were 3.7 and 12.1 months, respectively (Strickler et al., 2021). On the other hand, 2 of 4 patients treated with cabozantinib alone had SD and 2 had progressive disease (PD) as the best response (Jia et al., 2022). Of the 29 patients tested in (NCT02008383), 9 had MET amplification detected in their baseline (ctDNA), and 5 of those 9 patients were treated with cabozantinib and panitumumab combination therapy while 4 patients were treated with cabozantinib monotherapy. Intriguingly, of the 9 patients with MET amplification, 6 experienced clinical benefit; 1 PR and 3 SD in the cabozantinib and panitumumab combination group, and 2 SD in the cabozantinib alone group (Jia et al., 2022). The median PFS and median OS for MET-amplified patients was 3.7 and 5.7 months, respectively compared to median PFS and median OS of 2.5 and 12.1 months, respectively, in patients without MET amplification (Jia et al., 2022). Preclinically, cabozantinib was able to inhibit tumor growth, augment the immune response, and increase the immunogenicity in the human immune system (HIS)-BRGS mice implanted with MSS-CRC PDX tumors when combined with the anti-PD-1 mAb nivolumab (Lang et al., 2022). Lang et al. demonstrated that cabozantinib in combination with nivolumab significantly increased human T-cell infiltration with increased production of Granzyme B^+^ (GrB^+^) and TNFα^+^IFNγ^+^ double-producing CD4^+^ T cells among the tumor-infiltrating leukocytes (TILs), increased PD-L1 expression, along with HLA class I and II on tumor cells and decreased expression of TIM-3 on the CD4^+^ T cells, an indicative of a less exhausted T-cell pool in the HIS-BRGS mice (Lang et al., 2022). Lang et al. also reported that cabozantinib alone was able to increase the frequency of regulatory T cells (Tregs) among CD4^+^ T cells in TILs and upregulate the expression of PD-1 on the tumors of the HIS-BRGS mice (Lang et al., 2022). However, in contrast to Lang et al. findings, Kwilas and others reported that cabozantinib monotherapy significantly reduced the frequency of Tregs and MDSCs in the spleen (Kwilas et al., 2014) but significantly increased infiltrating CD8^+^ T cells of human carcinoembryonic antigen (CEA)-Tg C57/BL6 mice as well as significantly upregulated the expression of MHC-I molecules on tumor cells of the murine colon carcinoma cell line MC38-CEA, thus increasing the potential for antigen presentation and T-cell recognition of the tumor cells (Kwilas et al., 2014). Similarly, clinical data from the Phase II trial in patients with platinum-refractory metastatic urothelial carcinoma showed a reduction in the percentage of Tregs in the CD4^+^ T-cell population upon treatment with cabozantinib (Apolo et al., 2020). Also, cabozantinib can induce intratumoral neutrophil infiltration and generate a potent antitumor innate immune response resulting in rapid tumor eradication in CRC tumors in a PDX model and murine hepatocellular carcinoma (Esteban-Fabro et al., 2022) and prostate cancer models (Patnaik et al., 2017). Because cabozantinib can enhance both MHC class I and class II-mediated antigen presentation within the TME, it has demonstrated an immune regulatory effect with recruiting CD8^+^ T cells while reducing the presence of immunosuppressive cells (Tregs and MDSCs). Therefore, multiple ongoing clinical trials have combined cabozantinib with immune checkpoint inhibitors to generate a more permissive immune environment. For instance, patients with refractory metastatic pMMR/MSS CRC are being recruited for a Phase II study (NCT04963283) to assess the combination of cabozantinib and nivolumab. Also, cabozantinib is currently being evaluated in a Phase Ib study (NCT03170960) in combination with atezolizumab, anti-PD-L1 mAb in subjects with various tumor types, including CRC. Furthermore, evaluation of cabozantinib in combination with the anti-PD-L1 durvalumab with or without tremelimumab in advanced chemo-refractory pMMR/MSS gastroesophageal cancer and other gastrointestinal malignancies including CRC is currently ongoing in a Phase I/II trial (CAMILLA trial, NCT03539822). The results from the phase Ib part of the phase I/II trial (NCT03539822) demonstrated a favorable safety and suggested synergy between cabozantinib and durvalumab with ORR and DCR of 23.5% and 88.2%, respectively, and median PFS and median OS of 4.6 and 9.6 months, respectively among patients with CRC (*n* = 17) (Saeed et al., 2023). Interestingly, patients with a baseline PD-L1 combined positive score (CPS) of 5 or higher, low tumor CD68 and high CD4 protein levels had significantly improved ORR, PFS, and OS. Thus, PD-L1 CPS of 5 or more could be a predictive of response to this combination therapy. The recommended phase 2 dose (RP2D) for cabozantinib was 40 mg daily and the trial was expanded to a phase II multi-cohort, multi-center trial of 117 patients (Saeed et al., 2023). 36 heavily treated pMMR/MSS CRC patients were enrolled and received 40 mg QD of cabozantinib and 1,500 mg IV every 4 weeks (Q4W) of durvalumab in the phase II CRC cohort of the CAMILLA study. The preliminary interim results of the phase II CRC cohort of the CAMILLA study reported an ORR of 27.6%, confirmed partial response (PRc) of 21%, DCR of 86.2%, median PFS of 4.4 months, and median OS of 9.1 months among 29 patients evaluable for efficacy. Interestingly, of the 21% (7 out of 29 patients) who achieved PRc/SD > 6 months, one patient had KRAS G12V tumor mutation along with mutations in ARID1A and IDH1. Furthermore, in the RAS WT subgroup, the ORR (PRc) was 50.0%, the DCR was 83.3%, the median PFS was 6.3 months, and the median OS was not reached (Saeed et al., 2022). Furthermore, a phase I trial (NCT04868773) assessing the combination of cabozantinib with trifluridine/tipiracil (TAS-102) in mCRC patients is currently ongoing. **Tepotinib** is another nonselective c-MET inhibitor that is being investigated in a Phase II study (NCT04647838) in patients with solid cancers harboring c-MET amplification or exon 14 mutation who progressed after standard treatment for metastatic disease, including mCRC. A Phase II study (NCT04515394) also evaluated the preliminary antitumor activity of tepotinib in combination with cetuximab in patients with RAS/BRAF WT mCRC having acquired resistance to anti-EGFR due to MET amplification. However, the study was terminated early due to difficulties in identifying suitable participants for screening in the study.

## 6 Future directions and conclusion

The molecular subtyping for selecting the appropriate patient population for participation in clinical trials is also crucial to adequately test the validity of many targeting approaches. In clinical trials, the efficacy of signal transduction inhibitors may seem inadequate if evaluated in an unselected or improperly selected group of patients. Siemann et al. reported that most clinical trials testing c-MET inhibitors in cancer have yielded little benefit to patients and have not adequately tested the concept of c-MET pathway inhibition due to the lack of appropriate patient selection criteria (Hughes and Siemann, 2018). Siemann et al. also argued that c-MET mutation, c-MET amplification, or total c-MET expression, but not phospho-MET expression, have been used as markers for patient selection in these trials, which led to the inclusion of a large proportion of patients who will not benefit from the c-MET inhibitor, resulting in trial failure. Furthermore, because c-MET inhibitors are designed to reduce the phosphorylation of c-MET, c-MET phosphorylation is the most accurate biomarker for c-MET pathway activity and should be used as the inclusion criterion in clinical trials of c-MET inhibition (Hughes and Siemann, 2018).

Taken together, while the overall prognosis for CRC, especially in the metastatic setting, remains poor due to the intrinsic and acquired resistance to chemotherapy, targeted therapy, and immunotherapy, advances in our understanding of the resistance mechanisms of mCRC paved the way for new targeted biologic therapies and small molecules in both preclinical and clinical stages of development. Because mCRC is a heterogeneous disease resulting from the activation of different signaling pathways, combining chemotherapy with novel inhibitors targeting dysregulated pathways is necessary for a better outcome to prevent and overcome resistance. Combining standard chemotherapy with inhibitors of ABC drug transporters and combining EGFR-targeted therapy with inhibitors of c-MET can overcome drug resistance in preclinical models of advanced CRC, however; further clinical studies are needed to evaluate the clinical significance of these combination therapies in enhancing patient outcomes. Also, advances in diagnosis, gene detection methods such as ctDNA, and patient stratification based on the molecular profile of the tumors have enabled personalized care and led to improved outcomes for some subtypes of mCRC.

## References

[B1] Alketbi L. Al-Ali A. Talaat I. M. Hamid Q. Bajbouj K. (2023). The role of ATP-binding cassette subfamily A in colorectal cancer progression and resistance. Int. J. Mol. Sci. 24, 1344. 10.3390/ijms24021344 36674859 PMC9860967

[B2] Aoyama A. Katayama R. Oh-Hara T. Sato S. Okuno Y. Fujita N. (2014). Tivantinib (ARQ 197) exhibits antitumor activity by directly interacting with tubulin and overcomes ABC transporter-mediated drug resistance. Mol. Cancer Ther. 13, 2978–2990. 10.1158/1535-7163.MCT-14-0462 25313010

[B3] Apolo A. B. Nadal R. Tomita Y. Davarpanah N. N. Cordes L. M. Steinberg S. M. (2020). Cabozantinib in patients with platinum-refractory metastatic urothelial carcinoma: an open-label, single-centre, phase 2 trial. Lancet Oncol. 21, 1099–1109. 10.1016/S1470-2045(20)30202-3 32645282 PMC8236112

[B4] Arena S. Bellosillo B. Siravegna G. Martinez A. Canadas I. Lazzari L. (2015). Emergence of multiple EGFR extracellular mutations during cetuximab treatment in colorectal cancer. Clin. Cancer Res. 21, 2157–2166. 10.1158/1078-0432.CCR-14-2821 25623215

[B5] Arena S. Siravegna G. Mussolin B. Kearns J. D. Wolf B. B. Misale S. (2016). MM-151 overcomes acquired resistance to cetuximab and panitumumab in colorectal cancers harboring EGFR extracellular domain mutations. Sci. Transl. Med. 8, 324ra14. 10.1126/scitranslmed.aad5640 26843189

[B6] Baer M. R. George S. L. Dodge R. K. O'loughlin K. L. Minderman H. Caligiuri M. A. (2002). Phase 3 study of the multidrug resistance modulator PSC-833 in previously untreated patients 60 years of age and older with acute myeloid leukemia: cancer and Leukemia Group B Study 9720. Blood 100, 1224–1232. 10.1182/blood.v100.4.1224.h81602001224_1224_1232 12149202

[B7] Bagchi A. Haidar J. N. Eastman S. W. Vieth M. Topper M. Iacolina M. D. (2018). Molecular basis for necitumumab inhibition of EGFR variants associated with acquired cetuximab resistance. Mol. Cancer Ther. 17, 521–531. 10.1158/1535-7163.MCT-17-0575 29158469 PMC5925748

[B8] Baltschukat S. Engstler B. S. Huang A. Hao H. X. Tam A. Wang H. Q. (2019). Capmatinib (INC280) is active against models of non-small cell lung cancer and other cancer types with defined mechanisms of MET activation. Clin. Cancer Res. 25, 3164–3175. 10.1158/1078-0432.CCR-18-2814 30674502

[B9] Bardelli A. Corso S. Bertotti A. Hobor S. Valtorta E. Siravegna G. (2013). Amplification of the MET receptor drives resistance to anti-EGFR therapies in colorectal cancer. Cancer Discov. 3, 658–673. 10.1158/2159-8290.CD-12-0558 23729478 PMC4078408

[B10] Barlaam B. Anderton J. Ballard P. Bradbury R. H. Hennequin L. F. Hickinson D. M. (2013). Discovery of AZD8931, an equipotent, reversible inhibitor of signaling by EGFR, HER2, and HER3 receptors. ACS Med. Chem. Lett. 4, 742–746. 10.1021/ml400146c 24900741 PMC4027407

[B11] Basch E. Dueck A. C. Mitchell S. A. Mamon H. Weiser M. Saltz L. (2023). Patient-reported outcomes during and after treatment for locally advanced rectal cancer in the PROSPECT trial (alliance N1048). J. Clin. Oncol. 41, 3724–3734. 10.1200/JCO.23.00903 37270691 PMC10351948

[B12] Basilico C. Pennacchietti S. Vigna E. Chiriaco C. Arena S. Bardelli A. (2013). Tivantinib (ARQ197) displays cytotoxic activity that is independent of its ability to bind MET. Clin. Cancer Res. 19, 2381–2392. 10.1158/1078-0432.CCR-12-3459 23532890

[B13] Bendell J. C. Ervin T. J. Gallinson D. Singh J. Wallace J. A. Saleh M. N. (2013). Treatment rationale and study design for a randomized, double-blind, placebo-controlled phase II study evaluating onartuzumab (MetMAb) in combination with bevacizumab plus mFOLFOX-6 in patients with previously untreated metastatic colorectal cancer. Clin. Colorectal Cancer 12, 218–222. 10.1016/j.clcc.2013.04.001 23810377

[B14] Bendell J. C. Hochster H. Hart L. L. Firdaus I. Mace J. R. Mcfarlane J. J. (2017). A phase II randomized trial (GO27827) of first-line FOLFOX plus bevacizumab with or without the MET inhibitor onartuzumab in patients with metastatic colorectal cancer. Oncologist 22, 264–271. 10.1634/theoncologist.2016-0223 28209746 PMC5344636

[B15] Beretta G. L. Cassinelli G. Pennati M. Zuco V. Gatti L. (2017). Overcoming ABC transporter-mediated multidrug resistance: the dual role of tyrosine kinase inhibitors as multitargeting agents. Eur. J. Med. Chem. 142, 271–289. 10.1016/j.ejmech.2017.07.062 28851502

[B16] Bertotti A. Migliardi G. Galimi F. Sassi F. Torti D. Isella C. (2011). A molecularly annotated platform of patient-derived xenografts ("xenopatients") identifies HER2 as an effective therapeutic target in cetuximab-resistant colorectal cancer. Cancer Discov. 1, 508–523. 10.1158/2159-8290.CD-11-0109 22586653

[B17] Bertotti A. Papp E. Jones S. Adleff V. Anagnostou V. Lupo B. (2015). The genomic landscape of response to EGFR blockade in colorectal cancer. Nature 526, 263–267. 10.1038/nature14969 26416732 PMC4878148

[B18] Biller L. H. Schrag D. (2021). Diagnosis and treatment of metastatic colorectal cancer: a review. JAMA 325, 669–685. 10.1001/jama.2021.0106 33591350

[B19] Boccaccio C. Comoglio P. M. (2006). Invasive growth: a MET-driven genetic programme for cancer and stem cells. Nat. Rev. Cancer 6, 637–645. 10.1038/nrc1912 16862193

[B20] Boyer J. Mclean E. G. Aroori S. Wilson P. Mcculla A. Carey P. D. (2004). Characterization of p53 wild-type and null isogenic colorectal cancer cell lines resistant to 5-fluorouracil, oxaliplatin, and irinotecan. Clin. Cancer Res. 10, 2158–2167. 10.1158/1078-0432.ccr-03-0362 15041737

[B21] Brenner H. Kloor M. Pox C. P. (2014). Colorectal cancer. Lancet 383, 1490–1502. 10.1016/S0140-6736(13)61649-9 24225001

[B22] Bruijns S. R. Guly H. R. Bouamra O. Lecky F. Wallis L. A. (2014). The value of the difference between ED and prehospital vital signs in predicting outcome in trauma. Emerg. Med. J. 31, 579–582. 10.1136/emermed-2012-202271 23616498

[B23] Brunen D. Willems S. M. Kellner U. Midgley R. Simon I. Bernards R. (2013). TGF-β: an emerging player in drug resistance. Cell Cycle 12, 2960–2968. 10.4161/cc.26034 23974105 PMC3875670

[B24] Buettner R. Mora L. B. Jove R. (2002). Activated STAT signaling in human tumors provides novel molecular targets for therapeutic intervention. Clin. Cancer Res. 8, 945–954. 11948098

[B25] Burger H. Van Tol H. Brok M. Wiemer E. A. De Bruijn E. A. Guetens G. (2005). Chronic imatinib mesylate exposure leads to reduced intracellular drug accumulation by induction of the ABCG2 (BCRP) and ABCB1 (MDR1) drug transport pumps. Cancer Biol. Ther. 4, 747–752. 10.4161/cbt.4.7.1826 15970668

[B26] Calles A. Kwiatkowski N. Cammarata B. K. Ercan D. Gray N. S. Janne P. A. (2015). Tivantinib (ARQ 197) efficacy is independent of MET inhibition in non-small-cell lung cancer cell lines. Mol. Oncol. 9, 260–269. 10.1016/j.molonc.2014.08.011 25226813 PMC5528687

[B27] Ceballos M. P. Rigalli J. P. Ceré L. I. Semeniuk M. Catania V. A. Ruiz M. L. (2019). ABC transporters: regulation and association with multidrug resistance in hepatocellular carcinoma and colorectal carcinoma. Curr. Med. Chem. 26, 1224–1250. 10.2174/0929867325666180105103637 29303075

[B28] Cervantes A. Adam R. Roselló S. Arnold D. Normanno N. Taïeb J. (2023). Metastatic colorectal cancer: ESMO Clinical Practice Guideline for diagnosis, treatment and follow-up. Ann. Oncol. 34, 10–32. 10.1016/j.annonc.2022.10.003 36307056

[B29] Chalhoub N. Baker S. J. (2009). PTEN and the PI3-kinase pathway in cancer. Annu. Rev. Pathol. 4, 127–150. 10.1146/annurev.pathol.4.110807.092311 18767981 PMC2710138

[B30] Chaudhary N. Choudhary B. S. Shah S. G. Khapare N. Dwivedi N. Gaikwad A. (2021). Lipocalin 2 expression promotes tumor progression and therapy resistance by inhibiting ferroptosis in colorectal cancer. Int. J. Cancer 149, 1495–1511. 10.1002/ijc.33711 34146401

[B31] Chen X. Guan Z. Lu J. Wang H. Zuo Z. Ye F. (2018). Synergistic antitumor effects of cMet inhibitor in combination with anti-VEGF in colorectal cancer patient-derived xenograft models. J. Cancer 9, 1207–1217. 10.7150/jca.20964 29675102 PMC5907669

[B32] Chen Y. Zheng X. Wu C. (2021). The role of the tumor microenvironment and treatment strategies in colorectal cancer. Front. Immunol. 12, 792691. 10.3389/fimmu.2021.792691 34925375 PMC8674693

[B33] Chiavarina B. Costanza B. Ronca R. Blomme A. Rezzola S. Chiodelli P. (2021). Metastatic colorectal cancer cells maintain the TGFβ program and use TGFBI to fuel angiogenesis. Theranostics 11, 1626–1640. 10.7150/thno.51507 33408771 PMC7778592

[B34] Comoglio P. M. Giordano S. Trusolino L. (2008). Drug development of MET inhibitors: targeting oncogene addiction and expedience. Nat. Rev. Drug Discov. 7, 504–516. 10.1038/nrd2530 18511928

[B35] Corcoran R. B. Ebi H. Turke A. B. Coffee E. M. Nishino M. Cogdill A. P. (2012). EGFR-mediated re-activation of MAPK signaling contributes to insensitivity of BRAF mutant colorectal cancers to RAF inhibition with vemurafenib. Cancer Discov. 2, 227–235. 10.1158/2159-8290.CD-11-0341 22448344 PMC3308191

[B36] Cully M. You H. Levine A. J. Mak T. W. (2006). Beyond PTEN mutations: the PI3K pathway as an integrator of multiple inputs during tumorigenesis. Nat. Rev. Cancer 6, 184–192. 10.1038/nrc1819 16453012

[B37] Cuneo K. C. Mehta R. K. Kurapati H. Thomas D. G. Lawrence T. S. Nyati M. K. (2019). Enhancing the radiation response in KRAS mutant colorectal cancers using the c-met inhibitor crizotinib. Transl. Oncol. 12, 209–216. 10.1016/j.tranon.2018.10.005 30412912 PMC6226619

[B38] Cushman S. M. Jiang C. Hatch A. J. Shterev I. Sibley A. B. Niedzwiecki D. (2015). Gene expression markers of efficacy and resistance to cetuximab treatment in metastatic colorectal cancer: results from CALGB 80203 (Alliance). Clin. Cancer Res. 21, 1078–1086. 10.1158/1078-0432.CCR-14-2313 25520391 PMC4772749

[B39] Cuya S. M. Bjornsti M. A. Van Waardenburg R. (2017). DNA topoisomerase-targeting chemotherapeutics: what's new? Cancer Chemother. Pharmacol. 80, 1–14. 10.1007/s00280-017-3334-5 28528358 PMC9254606

[B40] Dai C. L. Tiwari A. K. Wu C. P. Su X. D. Wang S. R. Liu D. G. (2008). Lapatinib (Tykerb, GW572016) reverses multidrug resistance in cancer cells by inhibiting the activity of ATP-binding cassette subfamily B member 1 and G member 2. Cancer Res. 68, 7905–7914. 10.1158/0008-5472.CAN-08-0499 18829547 PMC2652245

[B41] Degirmenci U. Wang M. Hu J. (2020). Targeting aberrant RAS/RAF/MEK/ERK signaling for cancer therapy. Cells 9, 198. 10.3390/cells9010198 31941155 PMC7017232

[B42] Dekker E. Tanis P. J. Vleugels J. L. A. Kasi P. M. Wallace M. B. (2019). Colorectal cancer. Lancet 394, 1467–1480. 10.1016/S0140-6736(19)32319-0 31631858

[B43] Della Corte C. M. Fasano M. Papaccio F. Ciardiello F. Morgillo F. (2014). Role of HGF-MET signaling in primary and acquired resistance to targeted therapies in cancer. Biomedicines 2, 345–358. 10.3390/biomedicines2040345 28548075 PMC5344276

[B44] Delord J. P. Argiles G. Fayette J. Wirth L. Kasper S. Siena S. (2020). A phase 1b study of the MET inhibitor capmatinib combined with cetuximab in patients with MET-positive colorectal cancer who had progressed following anti-EGFR monoclonal antibody treatment. Invest. New Drugs 38, 1774–1783. 10.1007/s10637-020-00928-z 32410080

[B45] De Man F. M. Goey A. K. L. Van Schaik R. H. N. Mathijssen R. H. J. Bins S. (2018). Individualization of irinotecan treatment: a review of pharmacokinetics, pharmacodynamics, and pharmacogenetics. Clin. Pharmacokinet. 57, 1229–1254. 10.1007/s40262-018-0644-7 29520731 PMC6132501

[B46] Deng X. Ruan H. Zhang X. Xu X. Zhu Y. Peng H. (2020). Long noncoding RNA CCAL transferred from fibroblasts by exosomes promotes chemoresistance of colorectal cancer cells. Int. J. Cancer 146, 1700–1716. 10.1002/ijc.32608 31381140

[B47] De Vera A. A. Gupta P. Lei Z. Liao D. Narayanan S. Teng Q. (2019). Immuno-oncology agent IPI-549 is a modulator of P-glycoprotein (P-gp, MDR1, ABCB1)-mediated multidrug resistance (MDR) in cancer: *in vitro* and *in vivo* . Cancer Lett. 442, 91–103. 10.1016/j.canlet.2018.10.020 30391357 PMC6348084

[B48] Dienstmann R. Patnaik A. Garcia-Carbonero R. Cervantes A. Benavent M. Rosello S. (2015). Safety and activity of the first-in-class Sym004 anti-EGFR antibody mixture in patients with refractory colorectal cancer. Cancer Discov. 5, 598–609. 10.1158/2159-8290.CD-14-1432 25962717

[B49] Dienstmann R. Rodon J. Prat A. Perez-Garcia J. Adamo B. Felip E. (2014). Genomic aberrations in the FGFR pathway: opportunities for targeted therapies in solid tumors. Ann. Oncol. 25, 552–563. 10.1093/annonc/mdt419 24265351 PMC4433501

[B50] Dong H. Qiang Z. Chai D. Peng J. Xia Y. Hu R. (2020). Nrf2 inhibits ferroptosis and protects against acute lung injury due to intestinal ischemia reperfusion via regulating SLC7A11 and HO-1. Aging (Albany NY) 12, 12943–12959. 10.18632/aging.103378 32601262 PMC7377827

[B51] Dong S. Liang S. Cheng Z. Zhang X. Luo L. Li L. (2022). ROS/PI3K/Akt and Wnt/β-catenin signalings activate HIF-1α-induced metabolic reprogramming to impart 5-fluorouracil resistance in colorectal cancer. J. Exp. Clin. Cancer Res. 41, 15. 10.1186/s13046-021-02229-6 34998404 PMC8742403

[B52] Du J. He Y. Li P. Wu W. Chen Y. Ruan H. (2018). IL-8 regulates the doxorubicin resistance of colorectal cancer cells via modulation of multidrug resistance 1 (MDR1). Cancer Chemother. Pharmacol. 81, 1111–1119. 10.1007/s00280-018-3584-x 29693201

[B53] Elez E. Hendlisz A. Delaunoit T. Sastre J. Cervantes A. Varea R. (2016). Phase II study of necitumumab plus modified FOLFOX6 as first-line treatment in patients with locally advanced or metastatic colorectal cancer. Br. J. Cancer 114, 372–380. 10.1038/bjc.2015.480 26766738 PMC4815776

[B54] Eng C. Bessudo A. Hart L. L. Severtsev A. Gladkov O. Muller L. (2016). A randomized, placebo-controlled, phase 1/2 study of tivantinib (ARQ 197) in combination with irinotecan and cetuximab in patients with metastatic colorectal cancer with wild-type KRAS who have received first-line systemic therapy. Int. J. Cancer 139, 177–186. 10.1002/ijc.30049 26891420 PMC5071720

[B55] Eng C. Kim T. W. Bendell J. Argiles G. Tebbutt N. C. Di Bartolomeo M. (2019). Atezolizumab with or without cobimetinib versus regorafenib in previously treated metastatic colorectal cancer (IMblaze370): a multicentre, open-label, phase 3, randomised, controlled trial. Lancet Oncol. 20, 849–861. 10.1016/S1470-2045(19)30027-0 31003911

[B56] Esteban-Fabro R. Willoughby C. E. Pique-Gili M. Montironi C. Abril-Fornaguera J. Peix J. (2022). Cabozantinib enhances anti-PD1 activity and elicits a neutrophil-based immune response in hepatocellular carcinoma. Clin. Cancer Res. 28, 2449–2460. 10.1158/1078-0432.CCR-21-2517 35302601 PMC9167725

[B57] Fan Y. Tao T. Guo Z. Wah To K. K. Chen D. Wu S. (2022). Lazertinib improves the efficacy of chemotherapeutic drugs in ABCB1 or ABCG2 overexpression cancer cells *in vitro*, *in vivo*, and *ex vivo* . Mol. Ther. Oncolytics 24, 636–649. 10.1016/j.omto.2022.02.006 35284628 PMC8897717

[B58] Fang J. Y. Richardson B. C. (2005). The MAPK signalling pathways and colorectal cancer. Lancet Oncol. 6, 322–327. 10.1016/S1470-2045(05)70168-6 15863380

[B59] Fang Y. Zhong Q. Wang Y. Gu C. Liu S. Li A. (2020). CPEB3 functions as a tumor suppressor in colorectal cancer via JAK/STAT signaling. Aging (Albany NY) 12, 21404–21422. 10.18632/aging.103893 33146632 PMC7695424

[B60] Ferry D. R. Traunecker H. Kerr D. J. (1996). Clinical trials of P-glycoprotein reversal in solid tumours. Eur. J. Cancer 32A, 1070–1081. 10.1016/0959-8049(96)00091-3 8763349

[B61] Fojo A. T. Ueda K. Slamon D. J. Poplack D. G. Gottesman M. M. Pastan I. (1987). Expression of a multidrug-resistance gene in human tumors and tissues. Proc. Natl. Acad. Sci. U. S. A. 84, 265–269. 10.1073/pnas.84.1.265 2432605 PMC304184

[B62] Friedenberg W. R. Rue M. Blood E. A. Dalton W. S. Shustik C. Larson R. A. (2006). Phase III study of PSC-833 (valspodar) in combination with vincristine, doxorubicin, and dexamethasone (valspodar/VAD) versus VAD alone in patients with recurring or refractory multiple myeloma (E1A95): a trial of the Eastern Cooperative Oncology Group. Cancer 106, 830–838. 10.1002/cncr.21666 16419071

[B63] Fromme J. E. Schildhaus H. U. (2018). FGFR3 overexpression is a relevant alteration in colorectal cancer. Pathologe 39, 189–192. 10.1007/s00292-018-0504-0 30267148

[B64] Fu D. Pfannenstiel L. Demelash A. Phoon Y. P. Mayell C. Cabrera C. (2022). MCL1 nuclear translocation induces chemoresistance in colorectal carcinoma. Cell Death Dis. 13, 63. 10.1038/s41419-021-04334-y 35042842 PMC8766550

[B65] Fujii R. Seshimo A. Kameoka S. (2003). Relationships between the expression of thymidylate synthase, dihydropyrimidine dehydrogenase, and orotate phosphoribosyltransferase and cell proliferative activity and 5-fluorouracil sensitivity in colorectal carcinoma. Int. J. Clin. Oncol. 8, 72–78. 10.1007/s101470300013 12720098

[B66] Gallego R. Codony-Servat J. García-Albéniz X. Carcereny E. Longarón R. Oliveras A. (2009). Serum IGF-I, IGFBP-3, and matrix metalloproteinase-7 levels and acquired chemo-resistance in advanced colorectal cancer. Endocr. Relat. Cancer 16, 311–317. 10.1677/ERC-08-0250 19109398

[B67] Gan H. K. Millward M. Hua Y. Qi C. Sai Y. Su W. (2019). First-in-Human phase I study of the selective MET inhibitor, savolitinib, in patients with advanced solid tumors: safety, pharmacokinetics, and antitumor activity. Clin. Cancer Res. 25, 4924–4932. 10.1158/1078-0432.CCR-18-1189 30952639

[B68] Gandhi L. Harding M. W. Neubauer M. Langer C. J. Moore M. Ross H. J. (2007). A phase II study of the safety and efficacy of the multidrug resistance inhibitor VX-710 combined with doxorubicin and vincristine in patients with recurrent small cell lung cancer. Cancer 109, 924–932. 10.1002/cncr.22492 17285598

[B69] Gao H. L. Gupta P. Cui Q. Ashar Y. V. Wu Z. X. Zeng L. (2020). Sapitinib reverses anticancer drug resistance in colon cancer cells overexpressing the ABCB1 transporter. Front. Oncol. 10, 574861. 10.3389/fonc.2020.574861 33163405 PMC7581728

[B70] Gao R. Fang C. Xu J. Tan H. Li P. Ma L. (2019). LncRNA CACS15 contributes to oxaliplatin resistance in colorectal cancer by positively regulating ABCC1 through sponging miR-145. Arch. Biochem. Biophys. 663, 183–191. 10.1016/j.abb.2019.01.005 30639170

[B71] Giacomini K. M. Huang S. M. Tweedie D. J. Benet L. Z. Brouwer K. L. Chu X. (2010). Membrane transporters in drug development. Nat. Rev. Drug Discov. 9, 215–236. 10.1038/nrd3028 20190787 PMC3326076

[B72] Gogoi P. Kaur G. Singh N. K. (2022). Nanotechnology for colorectal cancer detection and treatment. World J. Gastroenterol. 28, 6497–6511. 10.3748/wjg.v28.i46.6497 36569271 PMC9782835

[B73] Grassilli E. Cerrito M. G. (2022). Emerging actionable targets to treat therapy-resistant colorectal cancers. Cancer Drug Resist 5, 36–63. 10.20517/cdr.2021.96 35582524 PMC8992594

[B74] Graves-Deal R. Bogatcheva G. Rehman S. Lu Y. Higginbotham J. N. Singh B. (2019). Broad-spectrum receptor tyrosine kinase inhibitors overcome *de novo* and acquired modes of resistance to EGFR-targeted therapies in colorectal cancer. Oncotarget 10, 1320–1333. 10.18632/oncotarget.26663 30863492 PMC6407678

[B75] Griffith D. A. Jarvis S. M. (1996). Nucleoside and nucleobase transport systems of mammalian cells. Biochim. Biophys. Acta 1286, 153–181. 10.1016/s0304-4157(96)00008-1 8982282

[B76] Griffith M. Mwenifumbo J. C. Cheung P. Y. Paul J. E. Pugh T. J. Tang M. J. (2013). Novel mRNA isoforms and mutations of uridine monophosphate synthetase and 5-fluorouracil resistance in colorectal cancer. Pharmacogenomics J. 13, 148–158. 10.1038/tpj.2011.65 22249354

[B77] Grothey A. Van Cutsem E. Sobrero A. Siena S. Falcone A. Ychou M. (2013). Regorafenib monotherapy for previously treated metastatic colorectal cancer (CORRECT): an international, multicentre, randomised, placebo-controlled, phase 3 trial. Lancet 381, 303–312. 10.1016/S0140-6736(12)61900-X 23177514

[B78] Gu Y. Chen Y. Wei L. Wu S. Shen K. Liu C. (2021). ABHD5 inhibits YAP-induced c-Met overexpression and colon cancer cell stemness via suppressing YAP methylation. Nat. Commun. 12, 6711. 10.1038/s41467-021-26967-5 34795238 PMC8602706

[B79] Guillén Díaz-Maroto N. Sanz-Pamplona R. Berdiel-Acer M. Cimas F. J. García E. Gonçalves-Ribeiro S. (2019). Noncanonical TGFβ pathway relieves the blockade of il1β/tgfβ-mediated crosstalk between tumor and stroma: TGFBR1 and TAK1 inhibition in colorectal cancer. Clin. Cancer Res. 25, 4466–4479. 10.1158/1078-0432.CCR-18-3957 30979739

[B80] Gymnopoulos M. Betancourt O. Blot V. Fujita R. Galvan D. Lieuw V. (2020). TR1801-ADC: a highly potent cMet antibody-drug conjugate with high activity in patient-derived xenograft models of solid tumors. Mol. Oncol. 14, 54–68. 10.1002/1878-0261.12600 31736230 PMC6944112

[B81] Hamaguchi K. Godwin A. K. Yakushiji M. O'dwyer P. J. Ozols R. F. Hamilton T. C. (1993). Cross-resistance to diverse drugs is associated with primary cisplatin resistance in ovarian cancer cell lines. Cancer Res. 53, 5225–5232. 8106143

[B82] Han H. Li Y. Qin W. Wang L. Yin H. Su B. (2022). miR-199b-3p contributes to acquired resistance to cetuximab in colorectal cancer by targeting CRIM1 via Wnt/β-catenin signaling. Cancer Cell Int. 22, 42. 10.1186/s12935-022-02460-x 35090460 PMC8796585

[B83] Han Y. Peng Y. Fu Y. Cai C. Guo C. Liu S. (2020). MLH1 deficiency induces cetuximab resistance in colon cancer via her-2/PI3K/AKT signaling. Adv. Sci. (Weinh) 7, 2000112. 10.1002/advs.202000112 32670759 PMC7341094

[B84] Hecht J. R. Trarbach T. Hainsworth J. D. Major P. Jager E. Wolff R. A. (2011). Randomized, placebo-controlled, phase III study of first-line oxaliplatin-based chemotherapy plus PTK787/ZK 222584, an oral vascular endothelial growth factor receptor inhibitor, in patients with metastatic colorectal adenocarcinoma. J. Clin. Oncol. 29, 1997–2003. 10.1200/JCO.2010.29.4496 21464406

[B85] Henriksson M. L. Edin S. Dahlin A. M. Oldenborg P. A. Oberg A. Van Guelpen B. (2011). Colorectal cancer cells activate adjacent fibroblasts resulting in FGF1/FGFR3 signaling and increased invasion. Am. J. Pathol. 178, 1387–1394. 10.1016/j.ajpath.2010.12.008 21356388 PMC3070577

[B86] Hirukawa A. Singh S. Wang J. Rennhack J. P. Swiatnicki M. Sanguin-Gendreau V. (2019). Reduction of global H3K27me(3) enhances HER2/ErbB2 targeted therapy. Cell Rep. 29, 249–257. 10.1016/j.celrep.2019.08.105 31597089

[B87] Houghton J. A. Houghton P. J. (1983). Elucidation of pathways of 5-fluorouracil metabolism in xenografts of human colorectal adenocarcinoma. Eur. J. Cancer Clin. Oncol. 19, 807–815. 10.1016/0277-5379(83)90013-5 6191988

[B88] Hsu H. C. Lapke N. Chen S. J. Lu Y. J. Jhou R. S. Yeh C. Y. (2018). PTPRT and PTPRD deleterious mutations and deletion predict bevacizumab resistance in metastatic colorectal cancer patients. Cancers (Basel) 10, 314. 10.3390/cancers10090314 30200630 PMC6162606

[B89] Hu F. Song D. Yan Y. Huang C. Shen C. Lan J. (2021). IL-6 regulates autophagy and chemotherapy resistance by promoting BECN1 phosphorylation. Nat. Commun. 12, 3651. 10.1038/s41467-021-23923-1 34131122 PMC8206314

[B90] Hu J. L. Wang W. Lan X. L. Zeng Z. C. Liang Y. S. Yan Y. R. (2019). CAFs secreted exosomes promote metastasis and chemotherapy resistance by enhancing cell stemness and epithelial-mesenchymal transition in colorectal cancer. Mol. Cancer 18, 91. 10.1186/s12943-019-1019-x 31064356 PMC6503554

[B91] Hu S. Dai H. Li T. Tang Y. Fu W. Yuan Q. (2016). Broad RTK-targeted therapy overcomes molecular heterogeneity-driven resistance to cetuximab via vectored immunoprophylaxis in colorectal cancer. Cancer Lett. 382, 32–43. 10.1016/j.canlet.2016.08.022 27569653

[B92] Hu Y. P. Patil S. B. Panasiewicz M. Li W. Hauser J. Humphrey L. E. (2008). Heterogeneity of receptor function in colon carcinoma cells determined by cross-talk between type I insulin-like growth factor receptor and epidermal growth factor receptor. Cancer Res. 68, 8004–8013. 10.1158/0008-5472.CAN-08-0280 18829558 PMC4472475

[B93] Hughes V. S. Siemann D. W. (2018). Have clinical trials properly assessed c-met inhibitors? Trends Cancer 4, 94–97. 10.1016/j.trecan.2017.11.009 29458966 PMC5824436

[B94] Humeniuk R. Menon L. G. Mishra P. J. Gorlick R. Sowers R. Rode W. (2009). Decreased levels of UMP kinase as a mechanism of fluoropyrimidine resistance. Mol. Cancer Ther. 8, 1037–1044. 10.1158/1535-7163.MCT-08-0716 19383847

[B95] Iacopetta B. (2003). TP53 mutation in colorectal cancer. Hum. Mutat. 21, 271–276. 10.1002/humu.10175 12619112

[B96] Ioffe D. Phull P. Dotan E. (2021). Optimal management of patients with advanced or metastatic cholangiocarcinoma: an evidence-based review. Cancer Manag. Res. 13, 8085–8098. 10.2147/CMAR.S276104 34737637 PMC8558827

[B97] Jacobs B. De Roock W. Piessevaux H. Van Oirbeek R. Biesmans B. De Schutter J. (2009). Amphiregulin and epiregulin mRNA expression in primary tumors predicts outcome in metastatic colorectal cancer treated with cetuximab. J. Clin. Oncol. 27, 5068–5074. 10.1200/JCO.2008.21.3744 19738126

[B98] Jia J. Howard L. Liu Y. Starr M. D. Brady J. C. Niedzwiecki D. (2022). Cabozantinib with or without Panitumumab for RAS wild-type metastatic colorectal cancer: impact of MET amplification on clinical outcomes and circulating biomarkers. Cancer Chemother. Pharmacol. 89, 413–422. 10.1007/s00280-022-04404-8 35171350

[B99] Jia X. Wang H. Li Z. Yan J. Guo Y. Zhao W. (2020). HER4 promotes the progression of colorectal cancer by promoting epithelial-mesenchymal transition. Mol. Med. Rep. 21, 1779–1788. 10.3892/mmr.2020.10974 32319604 PMC7057779

[B100] Jiang D. Li J. Li J. Wang M. Han C. Wang X. (2017). Combination of FGFR4 inhibitor Blu9931 and 5-fluorouracil effects on the biological characteristics of colorectal cancer cells. Int. J. Oncol. 51, 1611–1620. 10.3892/ijo.2017.4143 29048661

[B101] Jiang X. Stockwell B. R. Conrad M. (2021). Ferroptosis: mechanisms, biology and role in disease. Nat. Rev. Mol. Cell Biol. 22, 266–282. 10.1038/s41580-020-00324-8 33495651 PMC8142022

[B102] Jiang Y. Zhan H. (2020). Communication between EMT and PD-L1 signaling: new insights into tumor immune evasion. Cancer Lett. 468, 72–81. 10.1016/j.canlet.2019.10.013 31605776

[B103] Jing C. Ma R. Cao H. Wang Z. Liu S. Chen D. (2019). Long noncoding RNA and mRNA profiling in cetuximab-resistant colorectal cancer cells by RNA sequencing analysis. Cancer Med. 8, 1641–1651. 10.1002/cam4.2004 30848094 PMC6488152

[B104] Johnson S. M. Gulhati P. Rampy B. A. Han Y. Rychahou P. G. Doan H. Q. (2010). Novel expression patterns of PI3K/Akt/mTOR signaling pathway components in colorectal cancer. J. Am. Coll. Surg. 210, 767–776. 10.1016/j.jamcollsurg.2009.12.008 20421047 PMC2895913

[B105] Joosten S. P. J. Mizutani T. Spaargaren M. Clevers H. Pals S. T. (2019). MET signaling overcomes epidermal growth factor receptor inhibition in normal and colorectal cancer stem cells causing drug resistance. Gastroenterology 157, 1153–1155. 10.1053/j.gastro.2019.06.029 31255653

[B106] Joosten S. P. J. Spaargaren M. Clevers H. Pals S. T. (2020). Hepatocyte growth factor/MET and CD44 in colorectal cancer: partners in tumorigenesis and therapy resistance. Biochim. Biophys. Acta Rev. Cancer 1874, 188437. 10.1016/j.bbcan.2020.188437 32976979

[B107] Joosten S. P. J. Zeilstra J. Van Andel H. Mijnals R. C. Zaunbrecher J. Duivenvoorden A. a.M. (2017). MET signaling mediates intestinal crypt-villus development, regeneration, and adenoma formation and is promoted by stem cell CD44 isoforms. Gastroenterology 153, 1040–1053. 10.1053/j.gastro.2017.07.008 28716720

[B108] Kamoshida S. Matsuoka H. Ishikawa T. Maeda K. Shimomura R. Inada K. (2004). Immunohistochemical evaluation of thymidylate synthase (TS) and p16INK4a in advanced colorectal cancer: implication of TS expression in 5-FU-based adjuvant chemotherapy. Jpn. J. Clin. Oncol. 34, 594–601. 10.1093/jjco/hyh113 15591457

[B109] Kang K. A. Piao M. J. Kim K. C. Kang H. K. Chang W. Y. Park I. C. (2014). Epigenetic modification of Nrf2 in 5-fluorouracil-resistant colon cancer cells: involvement of TET-dependent DNA demethylation. Cell Death Dis. 5, e1183. 10.1038/cddis.2014.149 24743738 PMC4001304

[B110] Karamouzis M. V. Konstantinopoulos P. A. Papavassiliou A. G. (2009). Targeting MET as a strategy to overcome crosstalk-related resistance to EGFR inhibitors. Lancet Oncol. 10, 709–717. 10.1016/S1470-2045(09)70137-8 19573800

[B111] Karthikeyan S. Hoti S. L. (2015). Development of fourth generation ABC inhibitors from natural products: a novel approach to overcome cancer multidrug resistance. Anticancer Agents Med. Chem. 15, 605–615. 10.2174/1871520615666150113103439 25584696

[B112] Kathawala R. J. Gupta P. Ashby C. R. Jr. Chen Z. S. (2015). The modulation of ABC transporter-mediated multidrug resistance in cancer: a review of the past decade. Drug Resist Updat 18, 1–17. 10.1016/j.drup.2014.11.002 25554624

[B113] Kawakami H. Yonesaka K. (2016). HER3 and its ligand, heregulin, as targets for cancer therapy. Recent Pat. Anticancer Drug Discov. 11, 267–274. 10.2174/1574892811666160418123221 27086600

[B114] Kciuk M. Marciniak B. Kontek R. (2020). Irinotecan-still an important player in cancer chemotherapy: a comprehensive overview. Int. J. Mol. Sci. 21, 4919. 10.3390/ijms21144919 32664667 PMC7404108

[B115] Kearns J. D. Bukhalid R. Sevecka M. Tan G. Gerami-Moayed N. Werner S. L. (2015). Enhanced targeting of the EGFR network with MM-151, an oligoclonal anti-EGFR antibody therapeutic. Mol. Cancer Ther. 14, 1625–1636. 10.1158/1535-7163.MCT-14-0772 25911688

[B116] Kelly R. J. Draper D. Chen C. C. Robey R. W. Figg W. D. Piekarz R. L. (2011). A pharmacodynamic study of docetaxel in combination with the P-glycoprotein antagonist tariquidar (XR9576) in patients with lung, ovarian, and cervical cancer. Clin. Cancer Res. 17, 569–580. 10.1158/1078-0432.CCR-10-1725 21081657 PMC3071989

[B117] Khambata-Ford S. Garrett C. R. Meropol N. J. Basik M. Harbison C. T. Wu S. (2007). Expression of epiregulin and amphiregulin and K-ras mutation status predict disease control in metastatic colorectal cancer patients treated with cetuximab. J. Clin. Oncol. 25, 3230–3237. 10.1200/JCO.2006.10.5437 17664471

[B118] Kim S. A. Park H. Kim K. J. Kim J. W. Sung J. H. Nam M. (2022). Cetuximab resistance induced by hepatocyte growth factor is overcome by MET inhibition in KRAS, NRAS, and BRAF wild-type colorectal cancers. J. Cancer Res. Clin. Oncol. 148, 2995–3005. 10.1007/s00432-021-03872-4 34853888 PMC11801072

[B119] Koefoed K. Steinaa L. Soderberg J. N. Kjaer I. Jacobsen H. J. Meijer P. J. (2011). Rational identification of an optimal antibody mixture for targeting the epidermal growth factor receptor. MAbs 3, 584–595. 10.4161/mabs.3.6.17955 22123060 PMC3242845

[B120] Kornmann M. Schwabe W. Sander S. Kron M. Sträter J. Polat S. (2003). Thymidylate synthase and dihydropyrimidine dehydrogenase mRNA expression levels: predictors for survival in colorectal cancer patients receiving adjuvant 5-fluorouracil. Clin. Cancer Res. 9, 4116–4124. 14519634

[B121] Koustas E. Sarantis P. Theoharis S. Saetta A. A. Chatziandreou I. Kyriakopoulou G. (2019). Autophagy-related proteins as a prognostic factor of patients with colorectal cancer. Am. J. Clin. Oncol. 42, 767–776. 10.1097/COC.0000000000000592 31517637 PMC6766360

[B122] Krasteva N. Georgieva M. (2022). Promising therapeutic strategies for colorectal cancer treatment based on nanomaterials. Pharmaceutics 14, 1213. 10.3390/pharmaceutics14061213 35745786 PMC9227901

[B123] Kuang Y. H. Shen T. Chen X. Sodani K. Hopper-Borge E. Tiwari A. K. (2010). Lapatinib and erlotinib are potent reversal agents for MRP7 (ABCC10)-mediated multidrug resistance. Biochem. Pharmacol. 79, 154–161. 10.1016/j.bcp.2009.08.021 19720054 PMC2953260

[B124] Kwilas A. R. Ardiani A. Donahue R. N. Aftab D. T. Hodge J. W. (2014). Dual effects of a targeted small-molecule inhibitor (cabozantinib) on immune-mediated killing of tumor cells and immune tumor microenvironment permissiveness when combined with a cancer vaccine. J. Transl. Med. 12, 294. 10.1186/s12967-014-0294-y 25388653 PMC4236498

[B125] Lang J. Leal A. D. Marin-Jimenez J. A. Hartman S. J. Shulman J. Navarro N. M. (2022). Cabozantinib sensitizes microsatellite stable colorectal cancer to immune checkpoint blockade by immune modulation in human immune system mouse models. Front. Oncol. 12, 877635. 10.3389/fonc.2022.877635 36419897 PMC9676436

[B126] Lev A. Deihimi S. Shagisultanova E. Xiu J. Lulla A. R. Dicker D. T. (2017). Preclinical rationale for combination of crizotinib with mitomycin C for the treatment of advanced colorectal cancer. Cancer Biol. Ther. 18, 694–704. 10.1080/15384047.2017.1364323 28886275 PMC5663405

[B127] Li C. Singh B. Graves-Deal R. Ma H. Starchenko A. Fry W. H. (2017). Three-dimensional culture system identifies a new mode of cetuximab resistance and disease-relevant genes in colorectal cancer. Proc. Natl. Acad. Sci. U. S. A. 114, E2852–E2861. 10.1073/pnas.1618297114 28320945 PMC5389279

[B128] Li Q. Sun H. Luo D. Gan L. Mo S. Dai W. (2021). Lnc-RP11-536 K7.3/SOX2/HIF-1α signaling axis regulates oxaliplatin resistance in patient-derived colorectal cancer organoids. J. Exp. Clin. Cancer Res. 40, 348. 10.1186/s13046-021-02143-x 34740372 PMC8570024

[B129] Li X. Li X. Liao D. Wang X. Wu Z. Nie J. (2015). Elevated microRNA-23a expression enhances the chemoresistance of colorectal cancer cells with microsatellite instability to 5-fluorouracil by directly targeting ABCF1. Curr. Protein Pept. Sci. 16, 301–309. 10.2174/138920371604150429153309 25929864

[B130] Li X. Wu Z. Fu X. Han W. (2014). lncRNAs: insights into their function and mechanics in underlying disorders. Mutat. Res. Rev. Mutat. Res. 762, 1–21. 10.1016/j.mrrev.2014.04.002 25485593

[B131] Li Y. Gan Y. Liu J. Li J. Zhou Z. Tian R. (2022). Downregulation of MEIS1 mediated by ELFN1-AS1/EZH2/DNMT3a axis promotes tumorigenesis and oxaliplatin resistance in colorectal cancer. Signal Transduct. Target Ther. 7, 87. 10.1038/s41392-022-00902-6 35351858 PMC8964798

[B132] Li Z. N. Li M. Wang X. (2020a). Cancer type-dependent correlations between TP53 mutations and antitumor immunity. DNA Repair (Amst) 88, 102785. 10.1016/j.dnarep.2020.102785 32007736

[B133] Li Z. N. Zhao L. Yu L. F. Wei M. J. (2020b). BRAF and KRAS mutations in metastatic colorectal cancer: future perspectives for personalized therapy. Gastroenterol. Rep. (Oxf) 8, 192–205. 10.1093/gastro/goaa022 32665851 PMC7333923

[B134] Liao H. W. Hsu J. M. Xia W. Wang H. L. Wang Y. N. Chang W. C. (2015). PRMT1-mediated methylation of the EGF receptor regulates signaling and cetuximab response. J. Clin. Invest. 125, 4529–4543. 10.1172/JCI82826 26571401 PMC4665782

[B135] Liao W. Overman M. J. Boutin A. T. Shang X. Zhao D. Dey P. (2019). KRAS-IRF2 Axis drives immune suppression and immune therapy resistance in colorectal cancer. Cancer Cell 35, 559–572. 10.1016/j.ccell.2019.02.008 30905761 PMC6467776

[B136] Lieu C. H. Beeram M. Harb W. A. Kearns J. D. Sloss C. M. Nering R. (2015). Safety, pharmacology, and preliminary clinical activity of MM-151: an oligocolnal anti-EGFR theraputic in patients with cetuximab-resistant CRC and other refractory solid tumors. J. Clin. Oncol. 33, 647. 10.1200/jco.2015.33.3_suppl.647

[B137] Lin X. Stenvang J. Rasmussen M. H. Zhu S. Jensen N. F. Tarpgaard L. S. (2015). The potential role of Alu Y in the development of resistance to SN38 (Irinotecan) or oxaliplatin in colorectal cancer. BMC Genomics 16, 404. 10.1186/s12864-015-1552-y 25997618 PMC4440512

[B138] Linares J. Sallent-Aragay A. Badia-Ramentol J. Recort-Bascuas A. Méndez A. Manero-Rupérez N. (2023). Long-term platinum-based drug accumulation in cancer-associated fibroblasts promotes colorectal cancer progression and resistance to therapy. Nat. Commun. 14, 746. 10.1038/s41467-023-36334-1 36765091 PMC9918738

[B139] Lindskog E. B. Derwinger K. Gustavsson B. Falk P. Wettergren Y. (2014). Thymidine phosphorylase expression is associated with time to progression in patients with metastatic colorectal cancer. BMC Clin. Pathol. 14, 25. 10.1186/1472-6890-14-25 24936150 PMC4058433

[B140] Lippa M. S. Strockbine L. D. Le T. T. Branstetter D. G. Strathdee C. A. Holland P. M. (2007). Expression of anti-apoptotic factors modulates Apo2L/TRAIL resistance in colon carcinoma cells. Apoptosis 12, 1465–1478. 10.1007/s10495-007-0076-6 17440816

[B141] Liska D. Chen C. T. Bachleitner-Hofmann T. Christensen J. G. Weiser M. R. (2011). HGF rescues colorectal cancer cells from EGFR inhibition via MET activation. Clin. Cancer Res. 17, 472–482. 10.1158/1078-0432.CCR-10-0568 21098338 PMC3033451

[B142] Liu H. Liang Z. Zhou C. Zeng Z. Wang F. Hu T. (2021). Mutant KRAS triggers functional reprogramming of tumor-associated macrophages in colorectal cancer. Signal Transduct. Target Ther. 6, 144. 10.1038/s41392-021-00534-2 33833221 PMC8032794

[B143] Liu Q. Yu S. Zhao W. Qin S. Chu Q. Wu K. (2018). EGFR-TKIs resistance via EGFR-independent signaling pathways. Mol. Cancer 17, 53. 10.1186/s12943-018-0793-1 29455669 PMC5817859

[B144] Liu R. Li J. Xie K. Zhang T. Lei Y. Chen Y. (2013). FGFR4 promotes stroma-induced epithelial-to-mesenchymal transition in colorectal cancer. Cancer Res. 73, 5926–5935. 10.1158/0008-5472.CAN-12-4718 23943801

[B145] Liu Y. Zhang L. Chen X. Chen D. Shi X. Song J. (2022). The novel FGFR inhibitor F1-7 induces DNA damage and cell death in colon cells. Br. J. Cancer 127, 1014–1025. 10.1038/s41416-022-01878-4 35715638 PMC9470554

[B146] Longley D. B. Harkin D. P. Johnston P. G. (2003). 5-fluorouracil: mechanisms of action and clinical strategies. Nat. Rev. Cancer 3, 330–338. 10.1038/nrc1074 12724731

[B147] Longley D. B. Johnston P. G. (2005). Molecular mechanisms of drug resistance. J. Pathol. 205, 275–292. 10.1002/path.1706 15641020

[B148] Lotti F. Jarrar A. M. Pai R. K. Hitomi M. Lathia J. Mace A. (2013). Chemotherapy activates cancer-associated fibroblasts to maintain colorectal cancer-initiating cells by IL-17A. J. Exp. Med. 210, 2851–2872. 10.1084/jem.20131195 24323355 PMC3865474

[B149] Lu Y. Zhao X. Liu Q. Li C. Graves-Deal R. Cao Z. (2017). lncRNA MIR100HG-derived miR-100 and miR-125b mediate cetuximab resistance via Wnt/β-catenin signaling. Nat. Med. 23, 1331–1341. 10.1038/nm.4424 29035371 PMC5961502

[B150] Luo H. Zhang T. Cheng P. Li D. Ogorodniitchouk O. Lahmamssi C. (2020). Therapeutic implications of fibroblast growth factor receptor inhibitors in a combination regimen for solid tumors. Oncol. Lett. 20, 2525–2536. 10.3892/ol.2020.11858 32782571 PMC7400342

[B151] Ma S. L. Hu Y. P. Wang F. Huang Z. C. Chen Y. F. Wang X. K. (2014). Lapatinib antagonizes multidrug resistance-associated protein 1-mediated multidrug resistance by inhibiting its transport function. Mol. Med. 20, 390–399. 10.2119/molmed.2014.00059 25105301 PMC4212010

[B152] Ma Y. Yang Y. Wang F. Moyer M. P. Wei Q. Zhang P. (2016). Long non-coding RNA CCAL regulates colorectal cancer progression by activating Wnt/β-catenin signalling pathway via suppression of activator protein 2α. Gut 65, 1494–1504. 10.1136/gutjnl-2014-308392 25994219

[B153] Malinowsky K. Nitsche U. Janssen K. P. Bader F. G. Späth C. Drecoll E. (2014). Activation of the PI3K/AKT pathway correlates with prognosis in stage II colon cancer. Br. J. Cancer 110, 2081–2089. 10.1038/bjc.2014.100 24619078 PMC3992486

[B154] Manic S. Gatti L. Carenini N. Fumagalli G. Zunino F. Perego P. (2003). Mechanisms controlling sensitivity to platinum complexes: role of p53 and DNA mismatch repair. Curr. Cancer Drug Targets 3, 21–29. 10.2174/1568009033333727 12570658

[B155] Martens T. Schmidt N. O. Eckerich C. Fillbrandt R. Merchant M. Schwall R. (2006). A novel one-armed anti-c-Met antibody inhibits glioblastoma growth *in vivo* . Clin. Cancer Res. 12, 6144–6152. 10.1158/1078-0432.CCR-05-1418 17062691

[B156] Martins-Gomes C. Silva A. M. (2023). Natural products as a tool to modulate the activity and expression of multidrug resistance proteins of intestinal barrier. J. Xenobiot. 13, 172–192. 10.3390/jox13020014 37092502 PMC10123636

[B157] Maslankova J. Vecurkovska I. Rabajdova M. Katuchova J. Kicka M. Gayova M. (2022). Regulation of transforming growth factor-β signaling as a therapeutic approach to treating colorectal cancer. World J. Gastroenterol. 28, 4744–4761. 10.3748/wjg.v28.i33.4744 36156927 PMC9476856

[B158] Massoner P. Colleselli D. Matscheski A. Pircher H. Geley S. Jansen Dürr P. (2009). Novel mechanism of IGF-binding protein-3 action on prostate cancer cells: inhibition of proliferation, adhesion, and motility. Endocr. Relat. Cancer 16, 795–808. 10.1677/ERC-08-0175 19509068

[B159] Mathur A. Ware C. Davis L. Gazdar A. Pan B. S. Lutterbach B. (2014). FGFR2 is amplified in the NCI-H716 colorectal cancer cell line and is required for growth and survival. PLoS One 9, e98515. 10.1371/journal.pone.0098515 24968263 PMC4072591

[B160] Mccann C. Matveeva A. Mcallister K. Van Schaeybroeck S. Sessler T. Fichtner M. (2021). Development of a protein signature to enable clinical positioning of IAP inhibitors in colorectal cancer. Febs J. 288, 5374–5388. 10.1111/febs.15801 33660894

[B161] Merchant M. Ma X. Maun H. R. Zheng Z. Peng J. Romero M. (2013). Monovalent antibody design and mechanism of action of onartuzumab, a MET antagonist with anti-tumor activity as a therapeutic agent. Proc. Natl. Acad. Sci. U. S. A. 110, E2987–E2996. 10.1073/pnas.1302725110 23882082 PMC3740879

[B162] Meriggi F. Di Biasi B. Abeni C. Zaniboni A. (2009). Anti-EGFR therapy in colorectal cancer: how to choose the right patient. Curr. Drug Targets 10, 1033–1040. 10.2174/138945009789577891 19663767

[B163] Meschini S. Calcabrini A. Monti E. Del Bufalo D. Stringaro A. Dolfini E. (2000). Intracellular P-glycoprotein expression is associated with the intrinsic multidrug resistance phenotype in human colon adenocarcinoma cells. Int. J. Cancer 87, 615–628. 10.1002/1097-0215(20000901)87:5<615::aid-ijc1>3.3.co;2-w 10925353

[B164] Michel M. Kaps L. Maderer A. Galle P. R. Moehler M. (2021). The role of p53 dysfunction in colorectal cancer and its implication for therapy. Cancers (Basel) 13, 2296. 10.3390/cancers13102296 34064974 PMC8150459

[B165] Mizushima N. Komatsu M. (2011). Autophagy: renovation of cells and tissues. Cell 147, 728–741. 10.1016/j.cell.2011.10.026 22078875

[B166] Mohamad I. Barry A. Dawson L. Hosni A. (2022). Stereotactic body radiation therapy for colorectal liver metastases. Int. J. Hyperth. 39, 611–619. 10.1080/02656736.2021.1923836 35465818

[B167] Mohamed O. G. Salim A. A. Khalil Z. G. Elbanna A. H. Bernhardt P. V. Capon R. J. (2020). Chrysosporazines F-M: P-glycoprotein inhibitory phenylpropanoid piperazines from an Australian marine fish derived fungus, chrysosporium sp. CMB-F294. J. Nat. Prod. 83, 497–504. 10.1021/acs.jnatprod.9b01181 31975579

[B168] Mohn C. Kalayda G. V. Häcker H. G. Gütschow M. Metzger S. Jaehde U. (2010). Contribution of glutathione and MRP-mediated efflux to intracellular oxaliplatin accumulation. Int. J. Clin. Pharmacol. Ther. 48, 445–447. 10.5414/cpp48445 20557839

[B169] Montagut C. Argiles G. Ciardiello F. Poulsen T. T. Dienstmann R. Kragh M. (2018). Efficacy of Sym004 in patients with metastatic colorectal cancer with acquired resistance to anti-EGFR therapy and molecularly selected by circulating tumor DNA analyses: a phase 2 randomized clinical trial. JAMA Oncol. 4, e175245. 10.1001/jamaoncol.2017.5245 29423521 PMC5885274

[B170] Montagut C. Dalmases A. Bellosillo B. Crespo M. Pairet S. Iglesias M. (2012). Identification of a mutation in the extracellular domain of the Epidermal Growth Factor Receptor conferring cetuximab resistance in colorectal cancer. Nat. Med. 18, 221–223. 10.1038/nm.2609 22270724

[B171] Moore J. M. Bell E. L. Hughes R. O. Garfield A. S. (2023). ABC transporters: human disease and pharmacotherapeutic potential. Trends Mol. Med. 29, 152–172. 10.1016/j.molmed.2022.11.001 36503994

[B172] Morelli M. P. Overman M. J. Dasari A. Kazmi S. M. A. Mazard T. Vilar E. (2015). Characterizing the patterns of clonal selection in circulating tumor DNA from patients with colorectal cancer refractory to anti-EGFR treatment. Ann. Oncol. 26, 731–736. 10.1093/annonc/mdv005 25628445 PMC4374387

[B173] Morris V. K. Kennedy E. B. Baxter N. N. Benson A. B. 3rd Cercek A. Cho M. (2023). Treatment of metastatic colorectal cancer: ASCO guideline. J. Clin. Oncol. 41, 678–700. 10.1200/JCO.22.01690 36252154 PMC10506310

[B174] Mosca L. Pagano M. Borzacchiello L. Mele L. Russo A. Russo G. (2021). S-adenosylmethionine increases the sensitivity of human colorectal cancer cells to 5-fluorouracil by inhibiting P-glycoprotein expression and NF-κB activation. Int. J. Mol. Sci. 22, 9286. 10.3390/ijms22179286 34502219 PMC8431578

[B175] Munshi N. Jeay S. Li Y. Chen C. R. France D. S. Ashwell M. A. (2010). ARQ 197, a novel and selective inhibitor of the human c-Met receptor tyrosine kinase with antitumor activity. Mol. Cancer Ther. 9, 1544–1553. 10.1158/1535-7163.MCT-09-1173 20484018

[B176] Myint Z. W. Goel G. (2017). Role of modern immunotherapy in gastrointestinal malignancies: a review of current clinical progress. J. Hematol. Oncol. 10, 86. 10.1186/s13045-017-0454-7 28434400 PMC5402172

[B177] Napolitano S. Martini G. Martinelli E. Belli V. Parascandolo A. Laukkanen M. O. (2017a). Therapeutic efficacy of SYM004, a mixture of two anti-EGFR antibodies in human colorectal cancer with acquired resistance to cetuximab and MET activation. Oncotarget 8, 67592–67604. 10.18632/oncotarget.18749 28978055 PMC5620195

[B178] Napolitano S. Martini G. Martinelli E. Della Corte C. M. Morgillo F. Belli V. (2017b). Antitumor efficacy of triple monoclonal antibody inhibition of epidermal growth factor receptor (EGFR) with MM151 in EGFR-dependent and in cetuximab-resistant human colorectal cancer cells. Oncotarget 8, 82773–82783. 10.18632/oncotarget.19797 29137301 PMC5669927

[B179] Neiheisel A. Kaur M. Ma N. Havard P. Shenoy A. K. (2022). Wnt pathway modulators in cancer therapeutics: an update on completed and ongoing clinical trials. Int. J. Cancer 150, 727–740. 10.1002/ijc.33811 34536299

[B180] Nielsen D. L. Palshof J. A. Larsen F. O. Jensen B. V. Pfeiffer P. (2014). A systematic review of salvage therapy to patients with metastatic colorectal cancer previously treated with fluorouracil, oxaliplatin and irinotecan +/- targeted therapy. Cancer Treat. Rev. 40, 701–715. 10.1016/j.ctrv.2014.02.006 24731471

[B181] Nishida N. Yamashita S. Mimori K. Sudo T. Tanaka F. Shibata K. (2012). MicroRNA-10b is a prognostic indicator in colorectal cancer and confers resistance to the chemotherapeutic agent 5-fluorouracil in colorectal cancer cells. Ann. Surg. Oncol. 19, 3065–3071. 10.1245/s10434-012-2246-1 22322955

[B182] Noman M. Z. Parpal S. Van Moer K. Xiao M. Yu Y. Viklund J. (2020). Inhibition of Vps34 reprograms cold into hot inflamed tumors and improves anti-PD-1/PD-L1 immunotherapy. Sci. Adv. 6, eaax7881. 10.1126/sciadv.aax7881 32494661 PMC7190323

[B183] Ohishi T. Kaneko M. K. Yoshida Y. Takashima A. Kato Y. Kawada M. (2023). Current targeted therapy for metastatic colorectal cancer. Int. J. Mol. Sci. 24, 1702. 10.3390/ijms24021702 36675216 PMC9864602

[B184] Ou J. Miao H. Ma Y. Guo F. Deng J. Wei X. (2014). Loss of abhd5 promotes colorectal tumor development and progression by inducing aerobic glycolysis and epithelial-mesenchymal transition. Cell Rep. 9, 1798–1811. 10.1016/j.celrep.2014.11.016 25482557 PMC4268306

[B185] Owusu B. Y. Bansal N. Venukadasula P. K. Ross L. J. Messick T. E. Goel S. (2016). Inhibition of pro-HGF activation by SRI31215, a novel approach to block oncogenic HGF/MET signaling. Oncotarget 7, 29492–29506. 10.18632/oncotarget.8785 27121052 PMC5045412

[B186] Ozols R. F. Cunnion R. E. Klecker R. W. Jr. Hamilton T. C. Ostchega Y. Parrillo J. E. (1987). Verapamil and adriamycin in the treatment of drug-resistant ovarian cancer patients. J. Clin. Oncol. 5, 641–647. 10.1200/JCO.1987.5.4.641 3559654

[B187] Park S. Y. Lee C. J. Choi J. H. Kim J. H. Kim J. W. Kim J. Y. (2019). The JAK2/STAT3/CCND2 Axis promotes colorectal Cancer stem cell persistence and radioresistance. J. Exp. Clin. Cancer Res. 38, 399. 10.1186/s13046-019-1405-7 31511084 PMC6737692

[B188] Patnaik A. Swanson K. D. Csizmadia E. Solanki A. Landon-Brace N. Gehring M. P. (2017). Cabozantinib eradicates advanced murine prostate cancer by activating antitumor innate immunity. Cancer Discov. 7, 750–765. 10.1158/2159-8290.CD-16-0778 28274958 PMC5501767

[B189] Patnaik S. Anupriya A. (2019). Drugs targeting epigenetic modifications and plausible therapeutic strategies against colorectal cancer. Front. Pharmacol. 10, 588. 10.3389/fphar.2019.00588 31244652 PMC6563763

[B190] Paul M. D. Hristova K. (2019). The RTK interactome: overview and perspective on RTK heterointeractions. Chem. Rev. 119, 5881–5921. 10.1021/acs.chemrev.8b00467 30589534 PMC6918702

[B191] Pedersen M. W. Jacobsen H. J. Koefoed K. Hey A. Pyke C. Haurum J. S. (2010). Sym004: a novel synergistic anti-epidermal growth factor receptor antibody mixture with superior anticancer efficacy. Cancer Res. 70, 588–597. 10.1158/0008-5472.CAN-09-1417 20068188

[B192] Peters G. J. (2020). “Chapter 1 - drug resistance in colorectal cancer: general aspects,” in Drug resistance in colorectal cancer: molecular mechanisms and therapeutic strategies. Editors Cho, C. H. Hu T. (United States: Academic Press), 1–33.

[B193] Piao H. Y. Qu J. L. Liu Y. P. (2022). SOX8 promotes cetuximab resistance via HGF/MET bypass pathway activation in colorectal cancer. Cancer Chemother. Pharmacol. 89, 441–449. 10.1007/s00280-021-04378-z 35195773

[B194] Prager G. W. Taieb J. Fakih M. Ciardiello F. Van Cutsem E. Elez E. (2023). Trifluridine-tipiracil and bevacizumab in refractory metastatic colorectal cancer. N. Engl. J. Med. 388, 1657–1667. 10.1056/NEJMoa2214963 37133585

[B195] Pratt S. Shepard R. L. Kandasamy R. A. Johnston P. A. Perry W. 3rd Dantzig A. H. (2005). The multidrug resistance protein 5 (ABCC5) confers resistance to 5-fluorouracil and transports its monophosphorylated metabolites. Mol. Cancer Ther. 4, 855–863. 10.1158/1535-7163.MCT-04-0291 15897250

[B196] Raghav K. Morris V. Tang C. Morelli P. Amin H. M. Chen K. (2016). MET amplification in metastatic colorectal cancer: an acquired response to EGFR inhibition, not a *de novo* phenomenon. Oncotarget 7, 54627–54631. 10.18632/oncotarget.10559 27421137 PMC5342368

[B197] Ramesh P. Medema J. P. (2020). BCL-2 family deregulation in colorectal cancer: potential for BH3 mimetics in therapy. Apoptosis 25, 305–320. 10.1007/s10495-020-01601-9 32335811 PMC7244464

[B198] Ravindranathan P. Pasham D. Goel A. (2019). Oligomeric proanthocyanidins (OPCs) from grape seed extract suppress the activity of ABC transporters in overcoming chemoresistance in colorectal cancer cells. Carcinogenesis 40, 412–421. 10.1093/carcin/bgy184 30596962 PMC6514448

[B199] Ren J. Ding L. Zhang D. Shi G. Xu Q. Shen S. (2018). Carcinoma-associated fibroblasts promote the stemness and chemoresistance of colorectal cancer by transferring exosomal lncRNA H19. Theranostics 8, 3932–3948. 10.7150/thno.25541 30083271 PMC6071523

[B200] Riihimaki M. Hemminki A. Sundquist J. Hemminki K. (2016). Patterns of metastasis in colon and rectal cancer. Sci. Rep. 6, 29765. 10.1038/srep29765 27416752 PMC4945942

[B201] Rimassa L. Bozzarelli S. Pietrantonio F. Cordio S. Lonardi S. Toppo L. (2019). Phase II study of tivantinib and cetuximab in patients with KRAS wild-type metastatic colorectal cancer with acquired resistance to EGFR inhibitors and emergence of MET overexpression: lesson learned for future trials with EGFR/MET dual inhibition. Clin. Colorectal Cancer 18, 125–132. 10.1016/j.clcc.2019.02.004 30846365

[B202] Robles A. I. Jen J. Harris C. C. (2016). Clinical outcomes of TP53 mutations in cancers. Cold Spring Harb. Perspect. Med. 6, a026294. 10.1101/cshperspect.a026294 27449973 PMC5008065

[B203] Rodriguez P. C. Quiceno D. G. Zabaleta J. Ortiz B. Zea A. H. Piazuelo M. B. (2004). Arginase I production in the tumor microenvironment by mature myeloid cells inhibits T-cell receptor expression and antigen-specific T-cell responses. Cancer Res. 64, 5839–5849. 10.1158/0008-5472.CAN-04-0465 15313928

[B204] Romero-Garcia S. Prado-Garcia H. Carlos-Reyes A. (2020). Role of DNA methylation in the resistance to therapy in solid tumors. Front. Oncol. 10, 1152. 10.3389/fonc.2020.01152 32850327 PMC7426728

[B205] Rubin E. H. De Alwis D. P. Pouliquen I. Green L. Marder P. Lin Y. (2002). A phase I trial of a potent P-glycoprotein inhibitor, Zosuquidar.3HCl trihydrochloride (LY335979), administered orally in combination with doxorubicin in patients with advanced malignancies. Clin. Cancer Res. 8, 3710–3717. 12473580

[B206] Saeed A. Park R. Dai J. Al-Rajabi R. Kasi A. Baranda J. (2023). Cabozantinib plus durvalumab in advanced gastroesophageal cancer and other gastrointestinal malignancies: phase Ib CAMILLA trial results. Cell Rep. Med. 4, 100916. 10.1016/j.xcrm.2023.100916 36702123 PMC9975105

[B207] Saeed A. Park R. Dai J. Al-Rajabi R. M. D. T. Kasi A. Saeed A. (2022). Phase II trial of cabozantinib (Cabo) plus durvalumab (Durva) in chemotherapy refractory patients with advanced mismatch repair proficient/microsatellite stable (pMMR/MSS) colorectal cancer (CRC): CAMILLA CRC cohort results. J. Clin. Oncol. 40, 135. 10.1200/jco.2022.40.4_suppl.135

[B208] Saleh M. Cassier P. A. Eberst L. Naik G. Morris V. K. Pant S. (2020). Phase I study of ramucirumab plus merestinib in previously treated metastatic colorectal cancer: safety, preliminary efficacy, and pharmacokinetic findings. Oncologist 25, e1628–e1639. 10.1634/theoncologist.2020-0520 32537847 PMC7648328

[B209] Sanaei M. J. Baghery Saghchy Khorasani A. Pourbagheri-Sigaroodi A. Shahrokh S. Zali M. R. Bashash D. (2022). The PI3K/Akt/mTOR axis in colorectal cancer: oncogenic alterations, non-coding RNAs, therapeutic opportunities, and the emerging role of nanoparticles. J. Cell Physiol. 237, 1720–1752. 10.1002/jcp.30655 34897682

[B210] Sanchez-Martin F. J. Bellosillo B. Gelabert-Baldrich M. Dalmases A. Canadas I. Vidal J. (2016). The first-in-class anti-EGFR antibody mixture Sym004 overcomes cetuximab resistance mediated by EGFR extracellular domain mutations in colorectal cancer. Clin. Cancer Res. 22, 3260–3267. 10.1158/1078-0432.CCR-15-2400 26888827

[B211] Santarpia L. Lippman S. M. El-Naggar A. K. (2012). Targeting the MAPK-RAS-RAF signaling pathway in cancer therapy. Expert Opin. Ther. Targets 16, 103–119. 10.1517/14728222.2011.645805 22239440 PMC3457779

[B212] Santoro A. Rimassa L. Borbath I. Daniele B. Salvagni S. Van Laethem J. L. (2013). Tivantinib for second-line treatment of advanced hepatocellular carcinoma: a randomised, placebo-controlled phase 2 study. Lancet Oncol. 14, 55–63. 10.1016/S1470-2045(12)70490-4 23182627

[B213] Sartore-Bianchi A. Amatu A. Porcu L. Ghezzi S. Lonardi S. Leone F. (2019). HER2 positivity predicts unresponsiveness to EGFR-targeted treatment in metastatic colorectal cancer. Oncologist 24, 1395–1402. 10.1634/theoncologist.2018-0785 30952821 PMC6795149

[B214] Scagliotti G. Von Pawel J. Novello S. Ramlau R. Favaretto A. Barlesi F. (2015). Phase III multinational, randomized, double-blind, placebo-controlled study of tivantinib (ARQ 197) plus erlotinib versus erlotinib alone in previously treated patients with locally advanced or metastatic nonsquamous non-small-cell lung cancer. J. Clin. Oncol. 33, 2667–2674. 10.1200/JCO.2014.60.7317 26169611

[B215] Scartozzi M. Mandolesi A. Giampieri R. Pierantoni C. Loupakis F. Zaniboni A. (2010). Insulin-like growth factor 1 expression correlates with clinical outcome in K-RAS wild type colorectal cancer patients treated with cetuximab and irinotecan. Int. J. Cancer 127, 1941–1947. 10.1002/ijc.25193 20099280

[B216] Schwartz P. M. Moir R. D. Hyde C. M. Turek P. J. Handschumacher R. E. (1985). Role of uridine phosphorylase in the anabolism of 5-fluorouracil. Biochem. Pharmacol. 34, 3585–3589. 10.1016/0006-2952(85)90737-3 2996553

[B217] Scott A. J. Arcaroli J. J. Bagby S. M. Yahn R. Huber K. M. Serkova N. J. (2018). Cabozantinib exhibits potent antitumor activity in colorectal cancer patient-derived tumor xenograft models via autophagy and signaling mechanisms. Mol. Cancer Ther. 17, 2112–2122. 10.1158/1535-7163.MCT-17-0131 30026382 PMC6168336

[B218] Scott A. J. Basu Mallick A. Dotan E. Cohen S. J. Gold P. J. Hochster H. S. (2022). A phase II study investigating cabozantinib in patients with refractory metastatic colorectal cancer (AGICC 17CRC01). Cancer Res. Commun. 2, 1188–1196. 10.1158/2767-9764.CRC-22-0169 36969746 PMC10035393

[B219] Seager M. J. Jakobs T. F. Sharma R. A. Bandula S. (2021). Combination of ablation and embolization for intermediate-sized liver metastases from colorectal cancer: what can we learn from treating primary liver cancer? Diagn Interv. Radiol. 27, 677–683. 10.5152/dir.2021.20520 34318754 PMC8480946

[B220] Seiden M. V. Swenerton K. D. Matulonis U. Campos S. Rose P. Batist G. (2002). A phase II study of the MDR inhibitor biricodar (INCEL, VX-710) and paclitaxel in women with advanced ovarian cancer refractory to paclitaxel therapy. Gynecol. Oncol. 86, 302–310. 10.1006/gyno.2002.6762 12217752

[B221] Seligmann J. F. Elliott F. Richman S. D. Jacobs B. Hemmings G. Brown S. (2016). Combined epiregulin and amphiregulin expression levels as a predictive biomarker for panitumumab therapy benefit or lack of benefit in patients with RAS wild-type advanced colorectal cancer. JAMA Oncol. 2, 633–642. 10.1001/jamaoncol.2015.6065 26867820

[B222] Sethy C. Kundu C. N. (2021). 5-Fluorouracil (5-FU) resistance and the new strategy to enhance the sensitivity against cancer: implication of DNA repair inhibition. Biomed. Pharmacother. 137, 111285. 10.1016/j.biopha.2021.111285 33485118

[B223] Shi L. Li X. Wu Z. Li X. Nie J. Guo M. (2018). DNA methylation-mediated repression of miR-181a/135a/302c expression promotes the microsatellite-unstable colorectal cancer development and 5-FU resistance via targeting PLAG1. J. Genet. Genomics 45, 205–214. 10.1016/j.jgg.2018.04.003 29735329

[B224] Shi X. Kaller M. Rokavec M. Kirchner T. Horst D. Hermeking H. (2020). Characterization of a p53/miR-34a/CSF1R/STAT3 feedback loop in colorectal cancer. Cell Mol. Gastroenterol. Hepatol. 10, 391–418. 10.1016/j.jcmgh.2020.04.002 32304779 PMC7423584

[B225] Siegel R. L. Wagle N. S. Cercek A. Smith R. A. Jemal A. (2023). Colorectal cancer statistics, 2023. CA Cancer J. Clin. 73, 233–254. 10.3322/caac.21772 36856579

[B226] Slattery M. L. Lundgreen A. Kadlubar S. A. Bondurant K. L. Wolff R. K. (2013). JAK/STAT/SOCS-signaling pathway and colon and rectal cancer. Mol. Carcinog. 52, 155–166. 10.1002/mc.21841 22121102 PMC3430812

[B227] Smith A. D. Lu C. Payne D. Paschall A. V. Klement J. D. Redd P. S. (2020). Autocrine IL6-mediated activation of the STAT3-DNMT Axis silences the tnfα-RIP1 necroptosis pathway to sustain survival and accumulation of myeloid-derived suppressor cells. Cancer Res. 80, 3145–3156. 10.1158/0008-5472.CAN-19-3670 32554751 PMC7416440

[B228] Smith A. G. Macleod K. F. (2019). Autophagy, cancer stem cells and drug resistance. J. Pathol. 247, 708–718. 10.1002/path.5222 30570140 PMC6668344

[B229] Sodani K. Patel A. Kathawala R. J. Chen Z. S. (2012). Multidrug resistance associated proteins in multidrug resistance. Chin. J. Cancer 31, 58–72. 10.5732/cjc.011.10329 22098952 PMC3777468

[B230] Song E. K. Tai W. M. Messersmith W. A. Bagby S. Purkey A. Quackenbush K. S. (2015). Potent antitumor activity of cabozantinib, a c-MET and VEGFR2 inhibitor, in a colorectal cancer patient-derived tumor explant model. Int. J. Cancer 136, 1967–1975. 10.1002/ijc.29225 25242168 PMC4323738

[B231] Soong R. Shah N. Salto-Tellez M. Tai B. C. Soo R. A. Han H. C. (2008). Prognostic significance of thymidylate synthase, dihydropyrimidine dehydrogenase and thymidine phosphorylase protein expression in colorectal cancer patients treated with or without 5-fluorouracil-based chemotherapy. Ann. Oncol. 19, 915–919. 10.1093/annonc/mdm599 18245778 PMC2931808

[B232] Soundararajan R. Fradette J. J. Konen J. M. Moulder S. Zhang X. Gibbons D. L. (2019). Targeting the interplay between epithelial-to-mesenchymal-transition and the immune system for effective immunotherapy. Cancers (Basel) 11, 714. 10.3390/cancers11050714 31137625 PMC6562947

[B233] Spitzner M. Roesler B. Bielfeld C. Emons G. Gaedcke J. Wolff H. A. (2014). STAT3 inhibition sensitizes colorectal cancer to chemoradiotherapy *in vitro* and *in vivo* . Int. J. Cancer 134, 997–1007. 10.1002/ijc.28429 23934972 PMC7706351

[B234] Spoelstra E. C. Dekker H. Schuurhuis G. J. Broxterman H. J. Lankelma J. (1991). P-glycoprotein drug efflux pump involved in the mechanisms of intrinsic drug resistance in various colon cancer cell lines. Evidence for a saturation of active daunorubicin transport. Biochem. Pharmacol. 41, 349–359. 10.1016/0006-2952(91)90531-9 1671638

[B235] Stiff A. Trikha P. Mundy-Bosse B. Mcmichael E. Mace T. A. Benner B. (2018). Nitric oxide production by myeloid-derived suppressor cells plays a role in impairing fc receptor-mediated natural killer cell function. Clin. Cancer Res. 24, 1891–1904. 10.1158/1078-0432.CCR-17-0691 29363526 PMC7184799

[B236] Strickler J. H. Rushing C. N. Uronis H. E. Morse M. A. Niedzwiecki D. Blobe G. C. (2021). Cabozantinib and panitumumab for RAS wild-type metastatic colorectal cancer. Oncologist 26, 465–e917. 10.1002/onco.13678 33469991 PMC8176979

[B237] Strickler J. H. Yoshino T. Graham R. P. Siena S. Bekaii-Saab T. (2022). Diagnosis and treatment of ERBB2-positive metastatic colorectal cancer: a review. JAMA Oncol. 8, 760–769. 10.1001/jamaoncol.2021.8196 35238866

[B238] Sun C. Sun X. Shen B. (2017b). Molecular imaging of IGF-1R in cancer. Mol. Imaging 16, 1536012117736648. 10.1177/1536012117736648 29169312 PMC5703088

[B239] Sun C. Wang F. J. Zhang H. G. Xu X. Z. Jia R. C. Yao L. (2017a). miR-34a mediates oxaliplatin resistance of colorectal cancer cells by inhibiting macroautophagy via transforming growth factor-β/Smad4 pathway. World J. Gastroenterol. 23, 1816–1827. 10.3748/wjg.v23.i10.1816 28348487 PMC5352922

[B240] Sun P. Shi Y. Shi Y. (2023). Multivariate regression in conjunction with GA-BP for optimization of data processing of trace no gas flow in active pumping electronic nose. Sensors (Basel) 23, 1524. 10.3390/s23031524 36772572 PMC9919135

[B241] Suwaidan A. A. Lau D. K. Chau I. (2022). HER2 targeted therapy in colorectal cancer: new horizons. Cancer Treat. Rev. 105, 102363. 10.1016/j.ctrv.2022.102363 35228040

[B242] Tabernero J. Grothey A. Arnold D. De Gramont A. Ducreux M. O'dwyer P. (2022). MODUL cohort 2: an adaptable, randomized, signal-seeking trial of fluoropyrimidine plus bevacizumab with or without atezolizumab maintenance therapy for BRAF(wt) metastatic colorectal cancer. ESMO Open 7, 100559. 10.1016/j.esmoop.2022.100559 36029653 PMC9588902

[B243] Tabernero J. Grothey A. Van Cutsem E. Yaeger R. Wasan H. Yoshino T. (2021). Encorafenib plus cetuximab as a new standard of care for previously treated BRAF V600e-mutant metastatic colorectal cancer: updated survival results and subgroup analyses from the BEACON study. J. Clin. Oncol. 39, 273–284. 10.1200/JCO.20.02088 33503393 PMC8078423

[B244] Takahashi N. Yamada Y. Furuta K. Honma Y. Iwasa S. Takashima A. (2014). Serum levels of hepatocyte growth factor and epiregulin are associated with the prognosis on anti-EGFR antibody treatment in KRAS wild-type metastatic colorectal cancer. Br. J. Cancer 110, 2716–2727. 10.1038/bjc.2014.230 24800946 PMC4037834

[B245] Takano M. Sugiyama T. (2017). UGT1A1 polymorphisms in cancer: impact on irinotecan treatment. Pharmgenomics Pers. Med. 10, 61–68. 10.2147/PGPM.S108656 28280378 PMC5338934

[B246] Takeuchi H. Bilchik A. Saha S. Turner R. Wiese D. Tanaka M. (2003). c-MET expression level in primary colon cancer: a predictor of tumor invasion and lymph node metastases. Clin. Cancer Res. 9, 1480–1488. 12684423

[B247] Tam S. Y. Wu V. W. C. (2019). A review on the special radiotherapy techniques of colorectal cancer. Front. Oncol. 9, 208. 10.3389/fonc.2019.00208 31001474 PMC6454863

[B248] Tang Y. Zhou C. Li Q. Cheng X. Huang T. Li F. (2022). Targeting depletion of myeloid-derived suppressor cells potentiates PD-L1 blockade efficacy in gastric and colon cancers. Oncoimmunology 11, 2131084. 10.1080/2162402X.2022.2131084 36268178 PMC9578486

[B249] Tang Y. A. Chen Y. F. Bao Y. Mahara S. Yatim S. Oguz G. (2018). Hypoxic tumor microenvironment activates GLI2 via HIF-1α and TGF-β2 to promote chemoresistance in colorectal cancer. Proc. Natl. Acad. Sci. U. S. A. 115, E5990–E5999. 10.1073/pnas.1801348115 29891662 PMC6042102

[B250] Taniguchi K. Wada M. Kohno K. Nakamura T. Kawabe T. Kawakami M. (1996). A human canalicular multispecific organic anion transporter (cMOAT) gene is overexpressed in cisplatin-resistant human cancer cell lines with decreased drug accumulation. Cancer Res. 56, 4124–4129. 8797578

[B251] Thomas S. J. Snowden J. A. Zeidler M. P. Danson S. J. (2015). The role of JAK/STAT signalling in the pathogenesis, prognosis and treatment of solid tumours. Br. J. Cancer 113, 365–371. 10.1038/bjc.2015.233 26151455 PMC4522639

[B252] To K. K. Poon D. C. Wei Y. Wang F. Lin G. Fu L. W. (2015). Vatalanib sensitizes ABCB1 and ABCG2-overexpressing multidrug resistant colon cancer cells to chemotherapy under hypoxia. Biochem. Pharmacol. 97, 27–37. 10.1016/j.bcp.2015.06.034 26206183

[B253] Toor S. M. Syed Khaja A. S. El Salhat H. Bekdache O. Kanbar J. Jaloudi M. (2016). Increased levels of circulating and tumor-infiltrating granulocytic myeloid cells in colorectal cancer patients. Front. Immunol. 7, 560. 10.3389/fimmu.2016.00560 28008330 PMC5143474

[B254] Troiani T. Martinelli E. Napolitano S. Vitagliano D. Ciuffreda L. P. Costantino S. (2013). Increased TGF-α as a mechanism of acquired resistance to the anti-EGFR inhibitor cetuximab through EGFR-MET interaction and activation of MET signaling in colon cancer cells. Clin. Cancer Res. 19, 6751–6765. 10.1158/1078-0432.CCR-13-0423 24122793

[B255] Trumpi K. Emmink B. L. Prins A. M. Van Oijen M. G. Van Diest P. J. Punt C. J. (2015). ABC-transporter expression does not correlate with response to irinotecan in patients with metastatic colorectal cancer. J. Cancer 6, 1079–1086. 10.7150/jca.12606 26516354 PMC4615342

[B256] Valeri N. Gasparini P. Braconi C. Paone A. Lovat F. Fabbri M. (2010). MicroRNA-21 induces resistance to 5-fluorouracil by down-regulating human DNA MutS homolog 2 (hMSH2). Proc. Natl. Acad. Sci. U. S. A. 107, 21098–21103. 10.1073/pnas.1015541107 21078976 PMC3000294

[B257] Van Cutsem E. Cervantes A. Adam R. Sobrero A. Van Krieken J. H. Aderka D. (2016). ESMO consensus guidelines for the management of patients with metastatic colorectal cancer. Ann. Oncol. 27, 1386–1422. 10.1093/annonc/mdw235 27380959

[B258] Van Cutsem E. Eng C. Nowara E. Swieboda-Sadlej A. Tebbutt N. C. Mitchell E. (2014). Randomized phase Ib/II trial of rilotumumab or ganitumab with panitumumab versus panitumumab alone in patients with wild-type KRAS metastatic colorectal cancer. Clin. Cancer Res. 20, 4240–4250. 10.1158/1078-0432.CCR-13-2752 24919569 PMC4371780

[B259] Van Schaeybroeck S. Kalimutho M. Dunne P. D. Carson R. Allen W. Jithesh P. V. (2014). ADAM17-dependent c-MET-STAT3 signaling mediates resistance to MEK inhibitors in KRAS mutant colorectal cancer. Cell Rep. 7, 1940–1955. 10.1016/j.celrep.2014.05.032 24931611

[B260] Van Schaeybroeck S. Kyula J. N. Fenton A. Fenning C. S. Sasazuki T. Shirasawa S. (2011). Oncogenic Kras promotes chemotherapy-induced growth factor shedding via ADAM17. Cancer Res. 71, 1071–1080. 10.1158/0008-5472.CAN-10-0714 21148749 PMC3073126

[B261] Venukadasula P. K. Owusu B. Y. Bansal N. Ross L. J. Hobrath J. V. Bao D. (2016). Design and synthesis of nonpeptide inhibitors of hepatocyte growth factor activation. ACS Med. Chem. Lett. 7, 177–181. 10.1021/acsmedchemlett.5b00357 26985294 PMC4753539

[B262] Wang J. Tang Y. A. Xiao Q. Lee W. C. Cheng B. Niu Z. (2021b). Stromal induction of BRD4 phosphorylation results in chromatin remodeling and BET inhibitor resistance in colorectal cancer. Nat. Commun. 12, 4441. 10.1038/s41467-021-24687-4 34290255 PMC8295257

[B263] Wang J. Zhang Y. Song H. Yin H. Jiang T. Xu Y. (2021a). The circular RNA circSPARC enhances the migration and proliferation of colorectal cancer by regulating the JAK/STAT pathway. Mol. Cancer 20, 81. 10.1186/s12943-021-01375-x 34074294 PMC8167978

[B264] Wang L. Shen X. Chen G. Du J. (2022b). Drug resistance in colorectal cancer: from mechanism to clinic. Cancers (Basel) 14, 2928. 10.3390/cancers14122928 35740594 PMC9221177

[B265] Wang L. Zhao L. Lin Z. Yu D. Jin M. Zhou P. (2022a). Targeting DCLK1 overcomes 5-fluorouracil resistance in colorectal cancer through inhibiting CCAR1/β-catenin pathway-mediated cancer stemness. Clin. Transl. Med. 12, e743. 10.1002/ctm2.743 35522902 PMC9076011

[B266] Wang T. L. Diaz L. A. Jr. Romans K. Bardelli A. Saha S. Galizia G. (2004). Digital karyotyping identifies thymidylate synthase amplification as a mechanism of resistance to 5-fluorouracil in metastatic colorectal cancer patients. Proc. Natl. Acad. Sci. U. S. A. 101, 3089–3094. 10.1073/pnas.0308716101 14970324 PMC420348

[B267] Wang X. K. To K. K. Huang L. Y. Xu J. H. Yang K. Wang F. (2014). Afatinib circumvents multidrug resistance via dually inhibiting ATP binding cassette subfamily G member 2 *in vitro* and *in vivo* . Oncotarget 5, 11971–11985. 10.18632/oncotarget.2647 25436978 PMC4322967

[B268] Wang Y. Ding Y. Deng Y. Zheng Y. Wang S. (2020). Role of myeloid-derived suppressor cells in the promotion and immunotherapy of colitis-associated cancer. J. Immunother. Cancer 8, e000609. 10.1136/jitc-2020-000609 33051339 PMC7555106

[B269] Wang Y. J. Anderson M. G. Oleksijew A. Vaidya K. S. Boghaert E. R. Tucker L. (2017a). ABBV-399, a c-met antibody-drug conjugate that targets both MET-amplified and c-met-overexpressing tumors, irrespective of MET pathway dependence. Clin. Cancer Res. 23, 992–1000. 10.1158/1078-0432.CCR-16-1568 27573171

[B270] Wang Y. J. Zhang Y. K. Zhang G. N. Al Rihani S. B. Wei M. N. Gupta P. (2017b). Regorafenib overcomes chemotherapeutic multidrug resistance mediated by ABCB1 transporter in colorectal cancer: *in vitro* and *in vivo* study. Cancer Lett. 396, 145–154. 10.1016/j.canlet.2017.03.011 28302530 PMC5507680

[B271] Weekes J. Ho Y. H. Sebesan S. Ong K. Lam A. K. (2010). Irinotecan and colorectal cancer: the role of p53, VEGF-C and alpha-B-crystallin expression. Int. J. Colorectal Dis. 25, 907. 10.1007/s00384-009-0862-4 20012297

[B272] Wei F. Zhang T. Deng S. C. Wei J. C. Yang P. Wang Q. (2019). PD-L1 promotes colorectal cancer stem cell expansion by activating HMGA1-dependent signaling pathways. Cancer Lett. 450, 1–13. 10.1016/j.canlet.2019.02.022 30776481

[B273] Williams C. S. Bernard J. K. Demory Beckler M. Almohazey D. Washington M. K. Smith J. J. (2015). ERBB4 is over-expressed in human colon cancer and enhances cellular transformation. Carcinogenesis 36, 710–718. 10.1093/carcin/bgv049 25916654 PMC4572918

[B274] Winder T. Zhang W. Yang D. Ning Y. Bohanes P. Gerger A. (2010). Germline polymorphisms in genes involved in the IGF1 pathway predict efficacy of cetuximab in wild-type KRAS mCRC patients. Clin. Cancer Res. 16, 5591–5602. 10.1158/1078-0432.CCR-10-2092 20935157 PMC2982939

[B275] Wu C. Shao Y. Gu W. (2023). Immunotherapy combined with radiotherapy to reverse immunosuppression in microsatellite stable colorectal cancer. Clin. Transl. Oncol. 25, 1916–1928. 10.1007/s12094-023-03091-y 36717514

[B276] Wu S. Fu L. (2018). Tyrosine kinase inhibitors enhanced the efficacy of conventional chemotherapeutic agent in multidrug resistant cancer cells. Mol. Cancer 17, 25. 10.1186/s12943-018-0775-3 29455646 PMC5817862

[B277] Wu Y. Yakar S. Zhao L. Hennighausen L. Leroith D. (2002). Circulating insulin-like growth factor-I levels regulate colon cancer growth and metastasis. Cancer Res. 62, 1030–1035. 11861378

[B278] Xie T. Geng J. Wang Y. Wang L. Huang M. Chen J. (2017). FOXM1 evokes 5-fluorouracil resistance in colorectal cancer depending on ABCC10. Oncotarget 8, 8574–8589. 10.18632/oncotarget.14351 28051999 PMC5352423

[B279] Xie Y. H. Chen Y. X. Fang J. Y. (2020). Comprehensive review of targeted therapy for colorectal cancer. Signal Transduct. Target Ther. 5, 22. 10.1038/s41392-020-0116-z 32296018 PMC7082344

[B280] Yamamoto D. Oshima H. Wang D. Takeda H. Kita K. Lei X. (2022). Characterization of RNF43 frameshift mutations that drive Wnt ligand- and R-spondin-dependent colon cancer. J. Pathol. 257, 39–52. 10.1002/path.5868 35040131 PMC9314865

[B281] Yamamoto T. Miyoshi H. Kakizaki F. Maekawa H. Yamaura T. Morimoto T. (2020). Chemosensitivity of patient-derived cancer stem cells identifies colorectal cancer patients with potential benefit from FGFR inhibitor therapy. Cancers (Basel) 12, 2010. 10.3390/cancers12082010 32708005 PMC7465102

[B282] Yang Y. Jiang H. Li W. Chen L. Zhu W. Xian Y. (2020b). FOXM1/DVL2/Snail axis drives metastasis and chemoresistance of colorectal cancer. Aging (Albany NY) 12, 24424–24440. 10.18632/aging.202300 33291076 PMC7762457

[B283] Yang Y. Ni L. Imani S. Xiang Z. Hai R. Ding R. (2020a). Anlotinib suppresses colorectal cancer proliferation and angiogenesis via inhibition of AKT/ERK signaling cascade. Cancer Manag. Res. 12, 4937–4948. 10.2147/CMAR.S252181 32606981 PMC7321688

[B284] Yao J. F. Li X. J. Yan L. K. He S. Zheng J. B. Wang X. R. (2019). Role of HGF/c-Met in the treatment of colorectal cancer with liver metastasis. J. Biochem. Mol. Toxicol. 33, e22316. 10.1002/jbt.22316 30897285 PMC6617765

[B285] Ye P. Wang Y. Li R. Chen W. Wan L. Cai P. (2022). The HER family as therapeutic targets in colorectal cancer. Crit. Rev. Oncol. Hematol. 174, 103681. 10.1016/j.critrevonc.2022.103681 35462030

[B286] Yonesaka K. (2021). HER2-/HER3-Targeting antibody-drug conjugates for treating lung and colorectal cancers resistant to EGFR inhibitors. Cancers (Basel) 13, 1047. 10.3390/cancers13051047 33801379 PMC7958627

[B287] Yonesaka K. Satoh T. Ueda S. Yoshida T. Takeda M. Shimizu T. (2015). Circulating hepatocyte growth factor is correlated with resistance to cetuximab in metastatic colorectal cancer. Anticancer Res. 35, 1683–1689. 25750328

[B288] Young P. E. Womeldorph C. M. Johnson E. K. Maykel J. A. Brucher B. Stojadinovic A. (2014). Early detection of colorectal cancer recurrence in patients undergoing surgery with curative intent: current status and challenges. J. Cancer 5, 262–271. 10.7150/jca.7988 24790654 PMC3982039

[B289] Yu C. Luo D. Yu J. Zhang M. Zheng X. Xu G. (2022). Genome-wide CRISPR-cas9 knockout screening identifies GRB7 as a driver for MEK inhibitor resistance in KRAS mutant colon cancer. Oncogene 41, 191–203. 10.1038/s41388-021-02077-w 34718347 PMC8732282

[B290] Yu H. Rohan T. (2000). Role of the insulin-like growth factor family in cancer development and progression. J. Natl. Cancer Inst. 92, 1472–1489. 10.1093/jnci/92.18.1472 10995803

[B291] Yu J. Bai Y. Jin L. Zhang Z. Yang Y. (2023). A prospective long-term follow-up study: the application of circulating tumor cells analysis to guide adjuvant therapy in stage II colorectal cancer. Ann. Surg. Oncol. 30, 8495–8500. 10.1245/s10434-023-14168-x 37598121

[B292] Zhang H. W. Shi Y. Liu J. B. Wang H. M. Wang P. Y. Wu Z. J. (2021a). Cancer-associated fibroblast-derived exosomal microRNA-24-3p enhances colon cancer cell resistance to MTX by down-regulating CDX2/HEPH axis. J. Cell Mol. Med. 25, 3699–3713. 10.1111/jcmm.15765 33621425 PMC8051723

[B293] Zhang H. W. Zhang Y. Y. Chen Y. Wang J. Wang Q. Lu H. (2021b). TGF-Β signaling and resistance to cancer therapy. Front. Cell Dev. Biol. 9, 786728. 10.3389/fcell.2021.786728 34917620 PMC8669610

[B294] Zhang N. Yin Y. Xu S. J. Chen W. S. (2008). 5-Fluorouracil: mechanisms of resistance and reversal strategies. Molecules 13, 1551–1569. 10.3390/molecules13081551 18794772 PMC6244944

[B295] Zhang X. Chen Y. Hao L. Hou A. Chen X. Li Y. (2016). Macrophages induce resistance to 5-fluorouracil chemotherapy in colorectal cancer through the release of putrescine. Cancer Lett. 381, 305–313. 10.1016/j.canlet.2016.08.004 27514455

[B296] Zhang Y. Geng L. Talmon G. Wang J. (2015). MicroRNA-520g confers drug resistance by regulating p21 expression in colorectal cancer. J. Biol. Chem. 290, 6215–6225. 10.1074/jbc.M114.620252 25616665 PMC4358260

[B297] Zhang Y. Huang L. Shi H. Chen H. Tao J. Shen R. (2018). Ursolic acid enhances the therapeutic effects of oxaliplatin in colorectal cancer by inhibition of drug resistance. Cancer Sci. 109, 94–102. 10.1111/cas.13425 29034540 PMC5765292

[B298] Zhang Y. Wang X. (2020). Targeting the Wnt/β-catenin signaling pathway in cancer. J. Hematol. Oncol. 13, 165. 10.1186/s13045-020-00990-3 33276800 PMC7716495

[B299] Zhang Y. H. Luo D. D. Wan S. B. Qu X. J. (2020). S1PR2 inhibitors potently reverse 5-FU resistance by downregulating DPD expression in colorectal cancer. Pharmacol. Res. 155, 104717. 10.1016/j.phrs.2020.104717 32088343

[B300] Zhang Y. K. Wang Y. J. Lei Z. N. Zhang G. N. Zhang X. Y. Wang D. S. (2019). Regorafenib antagonizes BCRP-mediated multidrug resistance in colon cancer. Cancer Lett. 442, 104–112. 10.1016/j.canlet.2018.10.032 30392788 PMC8148022

[B301] Zhao H. Ming T. Tang S. Ren S. Yang H. Liu M. (2022). Wnt signaling in colorectal cancer: pathogenic role and therapeutic target. Mol. Cancer 21, 144. 10.1186/s12943-022-01616-7 35836256 PMC9281132

[B302] Zhou H. Lin C. Zhang Y. Zhang X. Zhang C. Zhang P. (2017). miR-506 enhances the sensitivity of human colorectal cancer cells to oxaliplatin by suppressing MDR1/P-gp expression. Cell Prolif. 50, e12341. 10.1111/cpr.12341 28217977 PMC6529089

[B303] Zhou Y. Zhang J. Wang K. Han W. Wang X. Gao M. (2020). Quercetin overcomes colon cancer cells resistance to chemotherapy by inhibiting solute carrier family 1, member 5 transporter. Eur. J. Pharmacol. 881, 173185. 10.1016/j.ejphar.2020.173185 32422185

